# Adhesion-mediated heterogeneous actin organization governs apoptotic cell extrusion

**DOI:** 10.1038/s41467-020-20563-9

**Published:** 2021-01-15

**Authors:** Anh Phuong Le, Jean-François Rupprecht, René-Marc Mège, Yusuke Toyama, Chwee Teck Lim, Benoît Ladoux

**Affiliations:** 1 Mechanobiology Institute, National University of Singapore, Singapore, Singapore; 2 National University of Singapore Graduate School of Integrative Sciences and Engineering, National University of Singapore, Singapore, Singapore; 3 Aix-Marseille Université, Université de Toulon, CNRS, CPT, Turing Centre for Living Systems, Marseille, France; 4 Université de Paris, CNRS, Institut Jacques Monod (IJM), Paris, France; 5 Department of Biological Sciences, National University of Singapore, Singapore, Singapore; 6 Department of Biomedical Engineering, National University of Singapore, Singapore, Singapore

**Keywords:** Cellular motility, Mechanotransduction, Apoptosis

## Abstract

Apoptotic extrusion is crucial in maintaining epithelial homeostasis. Current literature supports that epithelia respond to extrusion by forming a supracellular actomyosin purse-string in the neighbors. However, whether other actin structures could contribute to extrusion and how forces generated by these structures can be integrated are unknown. Here, we found that during extrusion, a heterogeneous actin network composed of lamellipodia protrusions and discontinuous actomyosin cables, was reorganized in the neighboring cells. The early presence of basal lamellipodia protrusion participated in both basal sealing of the extrusion site and orienting the actomyosin purse-string. The co-existence of these two mechanisms is determined by the interplay between the cell-cell and cell-substrate adhesions. A theoretical model integrates these cellular mechanosensitive components to explain why a dual-mode mechanism, which combines lamellipodia protrusion and purse-string contractility, leads to more efficient extrusion than a single-mode mechanism. In this work, we provide mechanistic insight into extrusion, an essential epithelial homeostasis process.

## Abstract (web summary)

Cell extrusion regulates monolayer cell density and is critical in maintaining epithelia integrity, which has implications in homeostasis, development, and cancer progression. Here the authors describe how monolayer integrate mechanical signals from tissue mechanics, cell-cell adhesion, cell-substrate adhesion and cytoskeleton coordinate cell extrusion.

## Introduction

Epithelia are cell sheets that act as a covering for most of the internal and external surfaces of the body. They play an important role as barriers. This function needs to be maintained, while epithelial cells are constantly challenged by the environment^1^. To face these environment challenges, epithelia are dynamics and have to deal constantly with cell renewal and cell extrusions, whose balance is key for epithelia homeostasis^2^. During cell extrusion, a cell embedded in an epithelial monolayer loses its apical or basal surface and is subsequently squeezed out of the monolayer by neighboring cells in the basal or luminal side, respectively. Cells can be extruded during apoptosis^3^, epithelial-mesenchymal transition (EMT)^4^, or precancerous cell invasion^5^, making cell extrusion a key player in the regulation of homeostatic pressure, i.e., pressure at which cell extrusion compensates cell division^6^, morphogenesis^7^, and tumor progression^8^. Cell extrusion leads to cell rearrangements and movements associated to the fluxes of cells and local stresses^9–11^. It relies on the establishment of mechanical coupling from the neighbors around the extruded cell, mediated by cell cytoskeletal components, and cell adhesion complexes. Cell–cell interactions and cell-matrix adhesions, which are known as mechanosensitive complexes^12,13^, appear as crucial relays to adapt the dynamic stress around the extrusion site across multiple cells^14,15^. The rearrangement of neighboring cells occurs concurrently with apoptotic cell death to expel the dying cell while maintaining intact monolayer^16^.

A well-described coordinated process of cell extrusion, referred to as actin purse-string contraction, is based on the emergence of contractile actomyosin rings, which are formed by the extruding cell itself^17,18^ and by the neighboring cells^18–20^. Both intrinsic and extrinsic apoptotic pathways can elicit extrusion, and caspase activation is essential to activate the Rho signaling pathway^3^, which promotes the formation and contraction of actomyosin rings. In mammalian cells, these rings were suggested to contract basolaterally and exert an upward force to expel the dying cell and close the gap left behind^18,19^. There are similarities drawn between the actomyosin contractile ring here and the supracellular purse-string in the wound healing mechanism^21–23^. Nevertheless, cell extrusion involves three-dimensional cell–cell junction reorganization at the interface of dying cell-neighboring interface^24–26^, while contractile purse-string in wound healing was described to exert forces in a single plane. Cells adjacent to the extrusion site partially lose polarity and reorganize their cell–cell junctions and actomyosin network^24,27^. Thus, the presence of the extruding cell instead of a void gap, which involves the additional roles of cell–cell junctions at the interface between dying cells and neighboring cells, distinguishes extrusion from wound healing. Therefore, how actomyosin purse-string could produce sufficient force in three dimensionality to drive extrusion in the polarized epithelia is still ambiguous.

Recently, we provided evidence that extrusion of apoptotic cells in epithelia with low packing density could occur with little purse-string involvement^10^. In this case, neighboring cells crawl towards extruding site and expel the dying cell via lamellipodia protrusions based on Rac1-regulated actin polymerization. This result is reminiscent of the wound healing process when heterogeneous actomyosin networks could concurrently contribute to gap closure with distinct force signature^28,29^. Nevertheless, whether the presence of lamellipodia impedes purse-string formation during extrusion was not clarified in previous studies. Also, as cells extend lamellipodia, cell-extracellular matrix (ECM) interactions through focal adhesions are remodeled, whether such dynamics of cell-substrate adhesions modulates actin reorganization during extrusion has not been understood so far.

In this study, we sought to determine the relative contributions of cell crawling and cell contractility to extrusion in relation with cell–cell and cell-substrate adhesions. We addressed these questions using MDCK cell culture as the model and a combination of techniques including laser-induced cell death, micropatterning, traction force microscopy, and molecular biology.

## Results

### Extrusion depends on both lamellipodia protrusion and purse-string

**Fig. 1:**
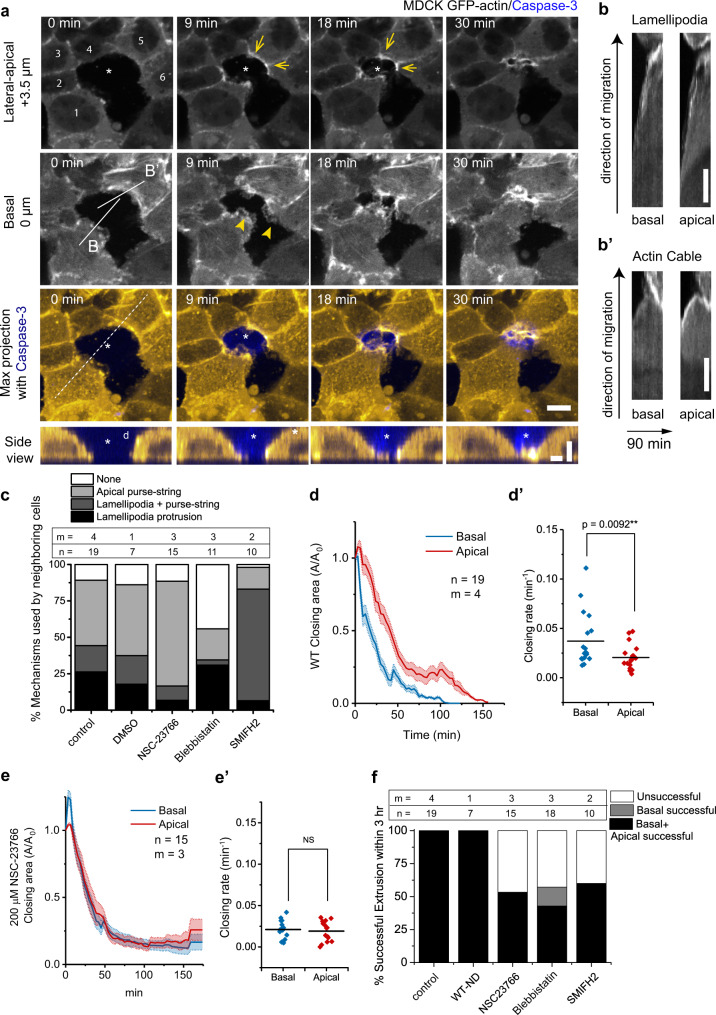
Epithelial extrusion depends on dual-mechanism mode: basal lamellipodia protrusion and purse-string formation. **a** Confocal time-lapse of a nonfluorescent WT MDCK cell (asterisk) extruding from monolayer with MDCK GFP-actin cells as neighbors. Arrows indicate actin cable, and arrowheads indicate lamellipodia protrusions. Colored panels represent maximum intensity projections from 0 μm (basal plane) to +9 μm. Scale bar = 10 μm. Bottom panel: side view along dashed line. Vertical scale bar = 5 µm. Horizontal scale bar = 10 µm. **b–b’** Kymographs performed along the connected line for lamellipodia (labeled as **b** in panel **a**) and along the connected line for actin cable (labeled as **b**’ in panel **a**), at basal and apical planes. Vertical scale bar = 5 µm. Time duration for all kymographs was 90 min. **c** Percentage of the mechanism chosen by neighboring cells in different pharmacological perturbation conditions: 200 µM NSC-23766 (Rac-inhibitor) and 50 µM (S)-nitro-Blebbistatin (SBB, Myosin activity inhibitor) and 50 µM SMIFH2 (Formin inhibitor). 0.1% DMSO was used as control for drug vehicle. n indicates number of extrusion events observed. m indicates number of independent experiments (experiments carried on different days). **d**, **e** Average relative area closure of basal and apical planes (defined as +4 µm from the basal plane), normalized to area at t = 0 min, as a function of time. Errors (SEM) are represented by shaded areas. Number of extrusion events *n* = 15, and number of independent experiments *m* = 3. **d’, e’** Closing rate derived by extracting the tangent of initial phase of the area closing curve. Middle lines: mean. Paired *t*-tests (two-tailed) were performed as comparison for closing speed between apical versus basal plane. Number of extrusion events and number of independent experiments are same as **c**. **f** Successful extrusion percentage for all conditions. Basal successful indicates complete basal closure but the cells were not extruded and new junctions between neighboring cells were not formed. Successful extrusion is indicated by basal successful + apical successful. Source data for **c**–**f** are provided as a Source Data file.

We monitored the actin organization at extruding cell-neighboring cell interfaces of laser-induced apoptotic cells from wild-type (WT) and fluorescently-labeled actin MDCK cells cocultured monolayers at high density (40–45 cells per 100 × 100 µm^2^). Thin actin-labeled lamellipodial protrusions were observed at the early time of extrusion at the basal plane of neighboring cells in both WT/GFP-actin MDCK cells (Fig. 1a, b) and WT/Ruby Lifeact MDCK cells mosaic cultures (Supplementary Movie 1). Thick bundles of actin indicating actomyosin cables were also formed in a nonuniform manner in neighboring cells at the apico-lateral plane (Fig. 1a, b’). These partial apical actomyosin cables were also observed during naturally occurring extrusion (Supplementary Fig. 2b). We validated the presence of lamellipodia by looking at cell extrusions in mosaic cultures of WT and MDCK expressing YFP-tagged p21 binding domain (PBD), a fluorescent biosensor of active Rac1 and Cdc42^30,31^ for both laser-induced cell death extrusions (Supplementary Fig. 1a, b) and naturally occurring extrusions (Supplementary Fig. 2a). Enhanced PBD signal in neighboring cells followed by directional cell edge movement towards the extrusion site coincided with the rise of caspase-3 signal in the extruding cell (marked by caspase-3 indicator DEVD-FMK), (Supplementary Fig. 1a, c, d, Supplementary Fig. 2a–a’), indicating that lamellipodial protrusion emerged in response to apoptosis. This directional movement of lamellipodia protrusion persists for a longer period than spontaneous lamellipodia fluctuations unrelated to apoptosis that usually exist in the monolayer (Supplementary Fig. 1e–e’). We observed similar processes at play for naturally occuring and laser-induced cell extrusions^10^. In the following, we will present data based on laser-induced extrusions to better control the initial conditions and ensure consistency between analyses.

To further evaluate the influence of cell protrusions versus actomyosin cable contractility, we looked at the evolution of the closing area defined by the edges of neighboring cells as a function of time in basal and apico-lateral planes. Complete basal sealing beneath the dying cells occurred in 30 min following laser stimulation (Fig. 1a), preceding apico-lateral area reduction (Fig. 1d–d’). MDCK monolayers treated with the Rac1 inhibitor NSC-23766, which inhibits lamellipodia formation^32^, displayed delayed basal area reduction (Fig. 1e–e’, Supplementary Fig. 3a), which was then concurrent with apical area closure. Rac1 inhibition also resulted in an increased proportion of neighboring cells forming actomyosin cables (Fig. 1c) as well as in a reduction of the number of successful extrusions (Fig. 1f). On the other hand, perturbing purse-string formation with myosin-II inhibitor—blebbistatin or formin inhibitor—SMIFH2 led to increased lamellipodia presence and faster closure rate of the basal plane (Supplementary Fig. 3b–d). Nevertheless, all these inhibitors reduced the number of successful extrusions (Fig. 1f and Supplementary Fig. 3b–d), indicating that extrusion was driven by combined effects of Rac-induced basal protrusions and apical contractions of the neighboring cells.

### Rac1-mediated lamellipodia protrusions resulted in heterogeneous apico-lateral actin organization

**Fig. 2:**
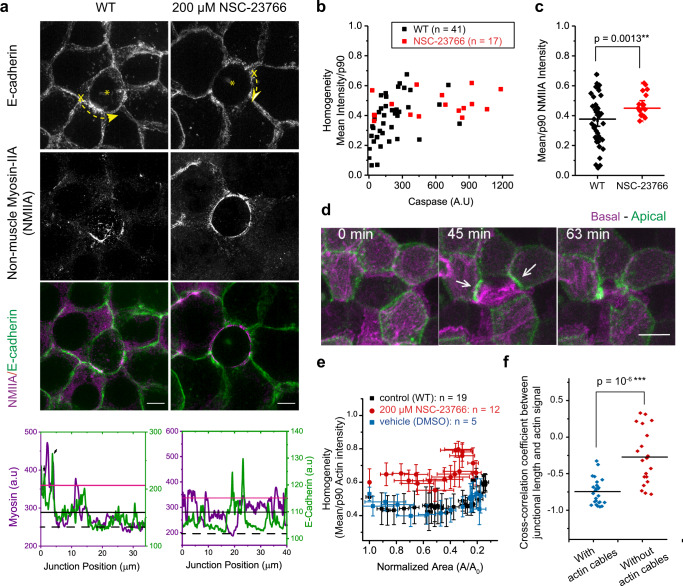
Inhomogeneous actomyosin cables were observed and persistent during extrusion. **a** Immunostaining of non-muscle myosin-IIA (NMIIA) of extruding cells (asterisks) in MDCK stably expressing GFP-E-cadherin (WT) monolayer versus Rac-inhibited monolayer. Images show maximal projection of z-stack from 2–6 µm above basal plane of SIM images. Scale bar = 10 µm. Bottom panel: line scan of 10-pixel width showing intensity profile along the junction between neighboring cells-extruding cell. Position 0 is indicated by X in the first panel. Dashed arrows indicate the direction the line scan was performed. **b** Relationship between NMIIA intensity inhomogeneity and fluorescence of caspase indicator. (Number of extrusion events: *n* = 41 for WT and *n* = 17 for Rac-inhibitor treatment. Number of independent experiments: m = 4 for WT and *m* = 2 for Rac-inhibitor treatment). Values closer to 1 indicate more homogeneous cables and closer to 0 indicates more nonuniform cables. **c** Inhomogeneity of actomyosin cables between WT versus Rac-inhibited monolayers by mean/p90 (normalized against the background intensity). Middle lines: mean. Error bars: SEM. Two-tailed unpaired *t*-test was performed. **d** Confocal time-lapse evolution of MDCK cell stably expressing Lifeact Ruby for actin undergoing extrusion. Basal plane (magenta) is superimposed with apical plane (green, 3.5 μm above the basal plane). Scale bar = 10 µm. T = 0 is defined as the time point right after laser induction. **e** Average inhomogeneity of actin cable (indicated by actin signals in live imaging) as a function of area normalized against value at time 0. Values close to 1 indicate more inhomogeneous actin cables and values close to 0 indicate more inhomogeneous actin cables. Error bars: SEM. Number of independent experiments: WT: *m* = 3; Rac-inhibitor (NSC-23766): *m* = 2; DMSO (drug control): *m* = 1. **f** Cross-correlation analysis between junctional length and average junctional intensity on individual junctions. (*n* = 26 for edges with cables and *n* = 20 for edges without cables in seven extrusions, three independent experiments). Middle lines: mean. Unpaired *t*-test (two-tailed) was performed. See more on Supplementary Fig. 5b. Source data in **a**–**f** are provided as a Source Data file.

In contrast to the expectation that a uniform, multicellular purse-string was prominent during extrusion as proposed in previous studies^33^, actin cables formed by surrounding cells were instead nonuniform (Fig. 1a, Supplementary Movie 1, Fig. 2a). We then questioned the contribution of such discontinuous actin cables to extrusion. We first quantified the inhomogeneity of the actomyosin cable by measuring the intensity of myosin-IIA immunostaining at extrusion sites (Fig. 2a) and compared this parameter with or without the Rac1 inhibitor. Caspase-3 was used as the surrogate marker for the initiation of apoptosis and stage of extrusion since the increase of caspase-3 levels as the function of time correlated with the reduction in apical closing area (Supplementary Fig. 4a–a’). The inhomogeneity in actomyosin II staining cable decreased with the intensity of caspase-3 (Fig. 2b; Supplementary Fig. 4b). Myosin distribution was only uniform at the final stage when the caspase-3 signal was maximum (Fig. 2b). We also quantified the inhomogeneity of actin distribution as a function of extrusion area and confirmed that inhomogeneous actomyosin cables persisted at neighboring-extruding cells interface until the end of extrusion (Fig. 2d–f; Supplementary Fig. 5a).

We rationalized that the purse-string may not need to span all cells to exert contractility: the actomyosin cables accumulated at each neighboring cell-extruding cell junctions could still contract. To test this hypothesis, we measured and cross-correlated the length of each junction as the function of time with its actin intensity over time (Fig.2f, Supplementary Fig. 5a–c). At the interfaces which form actin cables, the intensity of actin accumulation at these junctions correlates with junction’s shortening (Supplementary Fig. 5b). Such correlation for junctions that form cables was higher when compared with junctions that do not form actin cables (Supplementary Fig. 5b’–c, Fig. 2f). This result supported that contractile activity occurred at the individual junction level over the time-course of extrusion. This persistence of discontinuous purse-string is opposite from the view that a complete actomyosin ring is required for junctional reductions in neighboring cells.

**Fig. 3:**
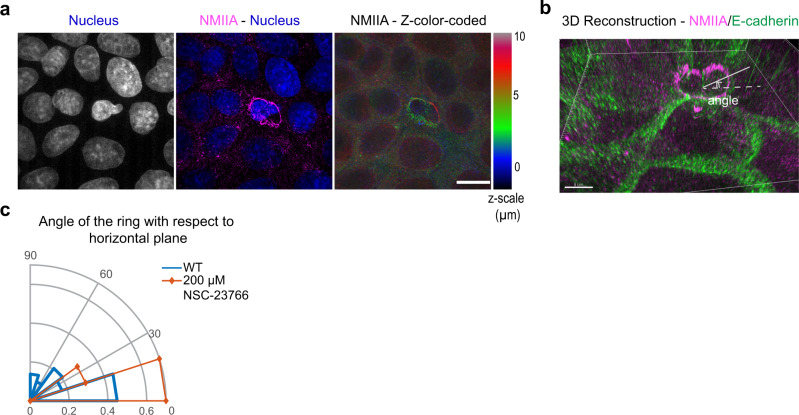
Localization of actomyosin cables in three-dimension. **a** Immunostaining of MDCK cells extruding from monolayer (extruding cell at the center), DAPI channel, Merged of DAPI and AlexaFluor-568 channel, Myosin staining color-coded according to the height of the monolayer. The contrasting localization of myosin around nucleus at two separated planes (~3 µm versus 7 µm at each side) suggested the partitioning of the purse-string into two rings which are tilted. Differential localization of actomyosin cables were observed in 34 extrusion events from 4 independent experiments. **b** 3D reconstruction for images in **a**. A circular fit was performed on each ring on 3D reconstruction images. The angle at which the circle formed with horizontal plane xy was defined as shown on the image. **c** Histogram of distribution of angles that the purse-string ring forms w.r.t horizontal plane. (WT: *n* = 32 rings in *m* = 19 extrusion events; NSC-23766 (Rac-inhibitor): *n* = 10 rings in *m* = 11 extrusion events). Source data are provided as a Source Data file.

As perturbing lamellipodia protrusion by NSC-23766 led to simultaneous reduction of basal and apical extrusion area (Fig. 1e–e’), we questioned the impact of lamellipodia formation on the assembly of actomyosin cables. We found that inhibiting lamellipodial extensions by NSC-23766 treatment led to a more uniform distribution of Myosin-II (Fig. 2a–e). Also, this uniform distribution of Myosin-II in NSC-23766-treated cells was independent of the caspase-3 levels (Fig. 3c). Even when actomyosin distribution became homogeneous and formed complete purse-strings around the apoptotic cell, these purse-strings were typically tilted in z over several µm (Fig. 3a, b). These actomyosin rings were tilted 31.6° ± 10.0° with respect to the horizontal plane, while they were typically more horizontally oriented 10.5° ± 2.8° in NSC-23766-treated monolayers (Fig. 3c). Therefore, lamellipodia protrusion might elicit the heterogeneous actomyosin organization during extrusion.

We postulated that the tilting of the full ring at the end stage of extrusion was due to two factors: nonuniform formation of contractile actin cables at apical levels and lamellipodia protrusions that delay myosin enrichment to form contractile cables. The representative example on Fig. 1a illustrates further this point. As cell extended lamellipodia (Fig. 1a, cell #1, top view and side view), the interface shared with its neighbors followed cell body extension and thus localized at the basal plane. This results in cell #2, #3, the side cell #4 connected to #3 being pulled to the basal plane. However, without lamellipodia protrusion, the rest of cell #4 remained at apical plane (18 min) when it started to form actomyosin cable. As a consequence, the actomyosin ring observed at late stages was partly connected to the basal part. To further support this model, we imaged cells co-expressing TdTomato F-Tractin and Myosin Regulatory Light Chain (MRLC) to distinguish actin at contractile cables and actin accumulated at protrusive membranes (Supplementary Fig. 6, Supplementary Movie 2). Actin protrusion at the basal sides was observed first, followed by myosin enrichment at the same plane and myosin reduction at 1–2 µm above, suggesting that basal protrusion helped to anchor actomyosin cables in the apical-to-basal direction.

These findings evidenced that lamellipodia protrusions preceded and caused heterogeneous patterns of actomyosin cables formation. As lamellipodial protrusion is stochastic, the resulted purse-string ring is heterogeneous, as observed. Early basal sealing was accomplished by lamellipodial protrusions, and extrusion is completed through apical closure by purse-string, which can either be partial or uniform. Failure of extrusion in the case of lamellipodia inhibition implied that such a sequence of events was more important than a single, uniform purse-string formation.

### Modulation of cell–cell junction strength dictates the dominant mechanism of extrusion

As partial actomyosin cables persisted during extrusion, we next questioned whether the presence of a full purse-string ring was dispensable for cell extrusion. In oncogenic extrusion, remodeling of E-cadherin followed by a redistribution of junctional tension was sufficient to drive extrusion without a prominent purse-string ring^24,26,34^. Adherens junctions have also been reported to mediate the rearrangement of cells surrounding the extrusion site^25^ and the formation of contractile actin cables^27^. Our experiments showed that junctional complexes between extruding and neighboring cells were remodeled. The reduction of junctional E-cadherin between extruding and neighboring cells was observed at the middle stage of extrusion (Supplementary Fig. 7a, 60 min, middle panel, white open arrows, Supplementary Fig. 7c, 78 min) in a nonuniform manner. Subsequently, inhomogeneous accumulations of active RhoA (Supplementary Fig. 7a, b) were observed. These sequences of events where molecular regulators of cellular tension assemble in a nonuniform fashion recall the partial actomyosin purse-string formation described so far.

**Fig. 4:**
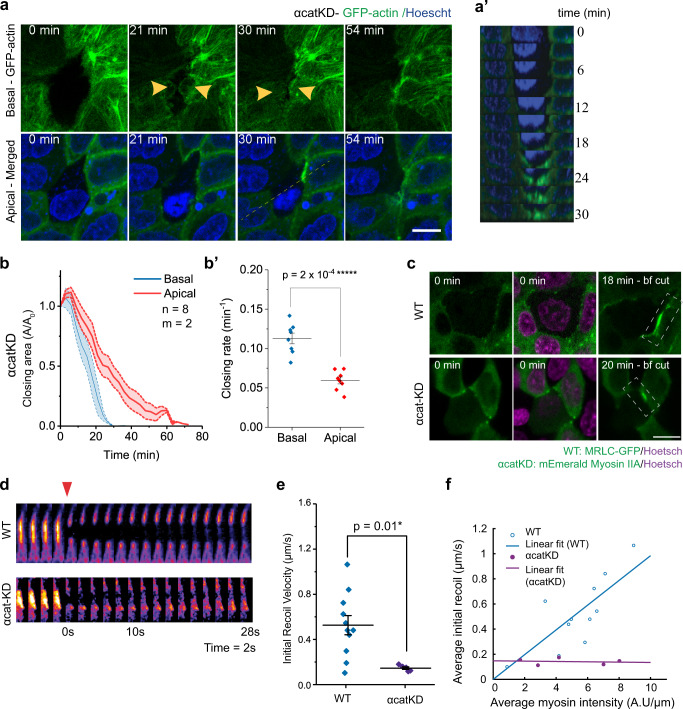
Reduction of cell–cell junction (CCJ) strength impairs purse-string formation and makes extrusion depend on lamellipodia protrusion. **a**–**a’** Time-lapse imaging of αcat- knocked-down MDCK cells (α-catKD-GFP-actin) expressing GFP-actin. The cell in the middle undergoes extrusion. **a** Top panels: basal plane, bottom panels: apical plane (+4 µm above basal plane). Scale bar = 10 µm. **a’** Side view along the dashed line, reconstructed from a Z-stack of 7 µm (0.5 µm slice). **b** Average relative closing area of basal versus apical planes (defined as +4 µm from the basal plane), normalized at t = 0 min for α-catKD-GFP-actin MDCK cells. Shaded area: SEM. *n* = 8 indicates number of extrusion from *m* = 2 independent experiments. **b’** Closing rate derived by extracting the tangent of initial phase of the area closing curve. Middle lines: mean. Error bars: SEM. Paired *t*-tests (two-tailed) were performed. **c**–**f** Laser ablation at CCJ during extrusion on WT MDCK cells expressing GFP-MRLC and αcatKD MDCK cells expressing m-Emerald Myosin-IIA. *N* = 11 for WT and *N* = 5 for αcatKD indicate number of laser ablation experiments. (**c**) Top view of myosin accumulation at CCJ. t = 0 min refers to the initiation of laser-induced extrusion. Images in the last panel (bf cut before cut) are at the time point laser ablation was performed on the cable. Dashed rectangles indicate the junction where laser ablation was performed. **d** Evolution of myosin signal from the rectangular area in **c** before and after laser ablation of cell–cell junctions. Enhanced myosin recruitment is shown in FIRE LUT. Arrowhead indicated the point at which laser ablation is performed. **e** Initial recoil velocity—which reflect junction tension—after ablation for WT versus αcatKD. Middle lines: mean. Error bars: SEM. Unpaired *t*-test (two-sided) was performed. Number of laser ablation experiments WT: *n* = 11 and αcatKD: *n* = 5. **f** Average initial recoil velocity as a function of average myosin accumulation prior to laser ablation. Middle lines: mean. Error bars: SEM. Linear regression was performed to fit. Source data for **b–f** are provided as a Source Data file.

We thus examined the contribution of cadherin-catenin complexes to cell extrusion by analyzing extrusion in α-catenin knocked-down (αcatKD) MDCK cells fully deficient for adherens junction (AJ) formation. We imaged actin distribution at extrusion sites (Fig. 4). Extrusion of these cells entirely engaged the lamellipodia-based mechanism (Fig. 4a, a’), and as a result, apical closure was strongly delayed compared to basal closure (Fig. 4b–b’). Occasionally, we observed the accumulation of actin at the apical plane for αcatKD cells facing the extruded cells (Fig. 4a, 30 min). To exclude the possibility that this accumulation of actin was part of functional contractile actomyosin cables, we performed laser ablation on these myosin- enriched interfaces and followed the associated junctional recoil (Fig. 4c–f). Laser ablation in αcatKD cells induced junctional recoil twice slower than the one observed in WT cells (Fig. 4d, e). Besides, initial recoil velocity of the ablated junction in αcatKD cells during extrusion was independent of junctional myosin accumulation, in contrast to the linear increase in WT case (Fig. 4f). These results indicated that weakened cell–cell junction (CCJ) in αcatKD cells caused impaired purse-string contractility.

**Fig. 5:**
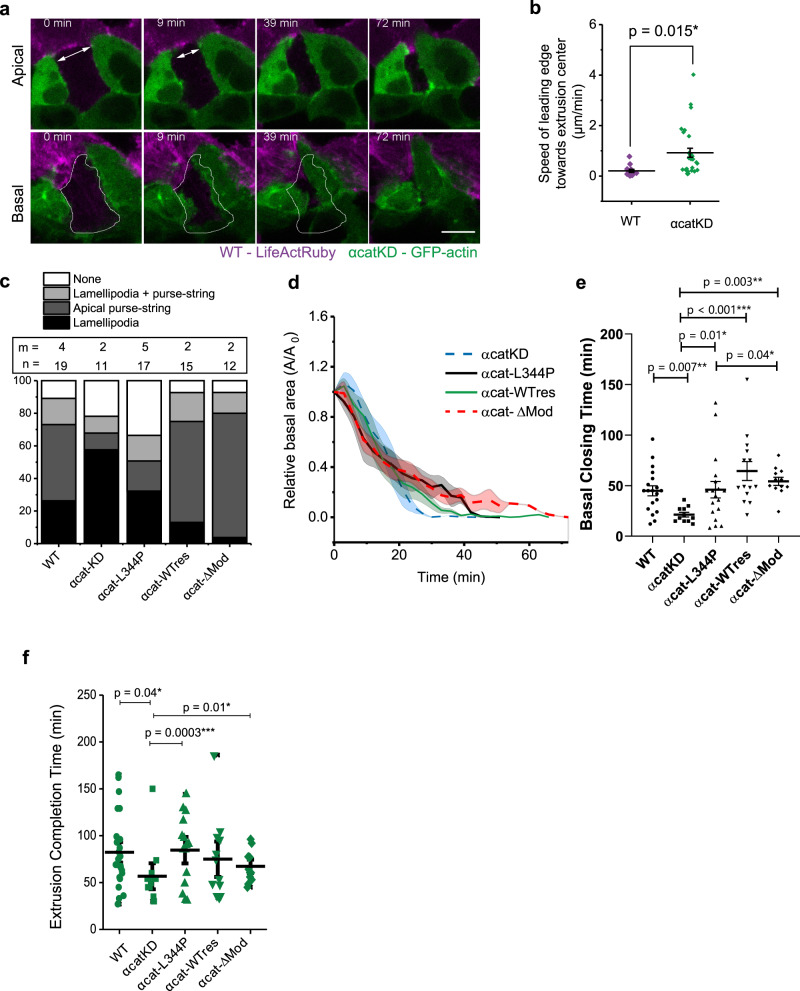
Tuning cell–cell junction strength by α-catenin could adjust the preferential mode of extrusion but not extrusion efficiency. **a**, **b** Laser-induced extrusion in mixed WT and αcatKD MDCK co-culture. The extruding cells are WT. **a** Top view of an example of extrusion from the mosaic monolayer. Scale bar = 10 µm. **b** Velocity towards the center of extrusion at basal side for each type of neighbor. Middle lines: mean. Error bars: SEM. *N* = 20 extrusion events. two-tailed, unpaired *t*-test was performed. **c** The contribution of mechanism at individual neighbors in single cell type cultures of different α-catenin mutant cells. αcat-KD MDCK cells with stable α-catenin knockdown. αcat-L344P MDCK α-catenin knockdown cells rescued with α-catenin-L344P mutant. αcat-WTres MDCK α-catenin knockdown cells rescued with wild-type GFP-α-catenin. αcat-ΔMod MDCK α-catenin knockdown cells rescued with α-catenin-ΔMod mutant. n number of extrusion events. m number of independent experiments. **d** Evolution with time of relative closure area changes at basal plane for cells expressing the different α-catenin mutants. Number of extrusion events and number of independent experiments are same in **c**. **e**: Mean basal closing times. One-way ANOVA (*p* = 0.0023**) followed by pair-wise comparison with Benjamini, Krieger and Yekutieli correction for false discovery rate was performed (*p* values are shown on the figure). Source data are provided as a Source Data file. **f** Mean extrusion completion time (both apical and basal area are closed). Middle lines: mean. Error bars: SEM. One-way ANOVA (*p* = 0.0006***) followed by pair-wise comparison with Benjamini, Krieger and Yekutieli correction for false discovery rate was performed. (*p* values are shown on the figure). Source data are provided as a Source Data file.

To artificially create a condition with different CCJ strength in between dying cell and neighboring cells, we cocultured WT and αcatKD MDCK cells and induced apoptosis in WT cells, which were surrounded by both WT and αcatKD cells. We found that neighboring αcatKD cells, with weakened CCJ engaged in extrusion could switch to lamellipodia-based protrusion and participate in basal closure (Fig. 5a, b), while neighboring WT cells formed contractile actin cable (Fig. 5a, top panel). In these mosaic monolayers, the αcatKD cells displayed faster basal cell edge movement towards the extrusion center than WT cells (Fig. 5b). This set of experiments confirmed that cells with weaker CCJ strength at the interface with the extruding cell used lamellipodia protrusion as the dominant mechanism.

We further proved that by varying CCJ strength using cells expressing various α-catenin mutants that could either reduce or enhance CCJ strength^35–37^, we could tune the mechanism of extrusion. The expression of these different α-catenin mutants into αcatKD cells (see Method section) rescued the contribution of actomyosin cables with different degrees (Fig. 5c, Supplementary Movie 3). αcat-L344P expressing cells, unable to recruit vinculin at CCJs despite being unable to form actin cables (Supplementary Movie 3), formed pronounced lamellipodial protrusions around extruded cells. In contrast, cells rescued with αcat-WT or αcat-ΔMod (constitutively recruiting vinculin) constructs formed more pronounced actin cables around extruded cells (Supplementary Movie 3). αcatKD MDCK cells, which formed lamellipodial protrusions closed the basal area faster than the other mutants, while αcat-ΔMod cells, which preferentially formed purse-string, displayed the most delayed basal area closure (Fig. 5d, e). This further validates that cadherin-mediated adhesions were crucial to modulate cell protrusion and actin cable activities during extrusion. Although either reducing or enhancing CCJ strength altered basal closure timing, it did not affect the duration of extrusion, as followed by apical closure timing (Fig. 5f). These data indicated that on substrate with homogeneous adhesion, monolayers with altered CCJ strength could adjust the relative contribution of lamellipodia protrusion/purse-string to successfully extrude dying cells.

### Basal lamellipodia protrusion controls cell extrusion via cell-substrate adhesion assembly

We further investigated the contribution of lamellipodia protrusion to ensure successful extrusion. The inhomogeneity of actomyosin cables at the apical plane was suggested to be the result of lamellipodia protrusion at the basal part, implicating the role of cell-matrix adhesion in the formation of these cables. Therefore, we aimed to decouple lamellipodia protrusion and actomyosin cable contractility by manipulating cell-substrate adhesion. We cultured MDCK monolayers on micropatterned substrates containing nonadhesive circular patches, and laser-induced apoptosis on cells sitting on top of these patches. We varied the size of the patches from 10 to 30 μm in diameter to cross-over subcellular and cellular dimensions and, thus, partially or entirely prevent cellular protrusions of the neighboring cells.

**Fig. 6:**
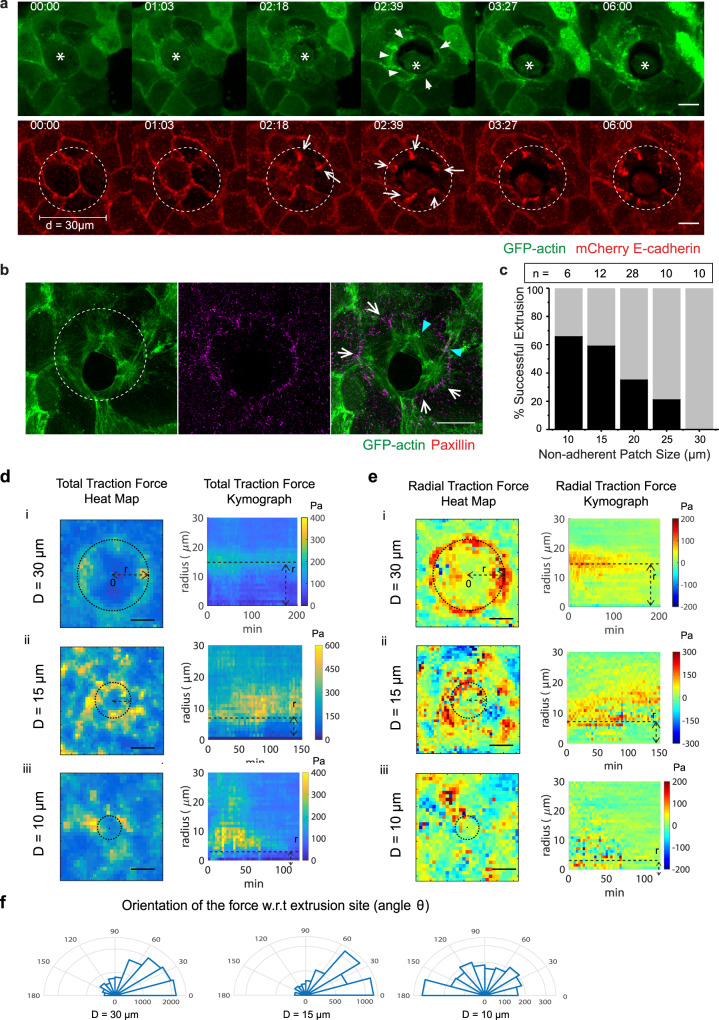
Substrate adherence influences the uniformity of actomyosin cables, extrusion efficiency, and forces contribution by purse-string/lamellipodia. **a** Confocal time-lapse imaging of laser-induced extrusion of a mCherry-E-cadherin, GFP-actin expressing MDCK cells on 30 µm nonadherent patch (edges indicated by dashed circles). The dying cell is marked with an asterisk. White arrowheads indicate actin purse-string and white arrows indicate reinforced CCJ. Scale bar = 10 µm. **b** SIM images of a fixed extruding GFP-actin expressing MDCK cell on nonadhesive patch. Cells were fixed and stained with anti-Paxillin antibodies. White arrow shows the radial actin cable connected with focal adhesion. Cyan arrowheads show cables anchoring between cell–cell junctions (indicated by actin converging points) and focal adhesion (indicated by paxillin staining). Scale bar = 10 µm. **c** Percentage of successful extrusion in function on nonadhesive patch size. n number of extrusion events. **d** Heatmap (left) and kymograph of average total traction magnitude (right) with respected to the center of the nonadhesive patches. Color bar indicated the force magnitude (Pa). Heatmap was the average of *m* = 50–60 time points over N extrusions, and kymograph was the average of N number of extrusion. (i) *N* = 5, (ii) *N* = 3, (iii) *N* = 4. **e** Heatmap (left) and kymograph of average radial traction force (right) with respected to the center of the nonadhesive patches as a function of time. Color bar indicated the force magnitude (Pa). +1 indicates forces pointing towards center (inwards) and −1 indicates forces pointing away from center (outwards). Heatmap was the average of m = 50–60 time points over N extrusions, and kymograph is the average of N number of extrusion. (i) *N* = 5, (ii) *N* = 3, (iii) *N* = 4. **f** Polar histogram showing the orientation of the force on D = 10, 15, and 30 µm nonadhesive patch. Angle θ is the radial angle of the force vector w.r.t the center of nonadhesive patch. *θ* < 90 shows inwards-pointing forces, 90 < *θ* < 180 shows outwards pointing forces. Source data for **c–f** are provided as the Source Data file.

Mechanical inhibition of cell protrusions by the nonadhesive patch (diameter = 30 µm) resulted in isotropic actin cable formation (Fig. 6a, Supplementary Movie 4). Continuous recruitment of actin cables to the purse-string appeared to pull on cell–cell junction (CCJ) as revealed by the accumulation of E-cadherin at the tips of surrounding CCJs (Fig. 6a, arrows, Supplementary Movie 4). The purse-string was also composed of radial cables, which emanated out of the continuous tangential cables, connecting to focal adhesion complexes at the edges of the nonadhesive patterns (Fig. 6b, white open arrow and Supplementary Fig. 8a, d) as previously observed during collective cell migration^38^. Some cables connect the focal adhesion with cell–cell junction (as visualized by enhanced actin at junction, pointed by a pair of cyan arrowheads in Fig. 5b). These observations revealed that the actin meshwork formed in response to extrusion was not only supported by multicellular actomyosin cable anchored across cell–cell junctions but also emerged from cables connected to cell-substrate adhesions. Laser ablation performed on these radial cables resulted in the recoil of both the purse-string (Supplementary Fig. 8a, b, b’, initial recoil velocity = 0.13 ± 0.01 µm s^−1^, Supplementary Movie 5) and the rear of the cell away from extrusion site (Supplementary Fig. 8a, c, c’, initial recoil velocity = 0.07 ± 0.01 µm s^−1^) indicating that these cables are under tension.

In agreement with our data on pharmacological inhibition of lamellipodia (Fig. 1e–e’), the uniform purse-string alone was not sufficient to extrude apoptotic cells at 30*-*μm diameter nonadhesive patches (Fig. 6a, Supplementary Movie 4). The dying cell detached from its neighbors without the simultaneous sealing activity from the neighbors (Fig. 6a, Supplementary Fig. 9a, Supplementary Movie 4). To investigate the extent to which the lack of substrate adhesion impeded extrusion we varied the nonadhesive patch size (Fig. 6c and Supplementary Fig. 9). A monotonic trend was observed between the patch size and extrusion efficiency (Fig. 6c, Supplementary Fig. 9b-b’), supporting that by forming nascent focal adhesions, protrusions from neighboring cells control the formation of purse-string to extrude the dying cells.

### Deconstructing the mechanical forces of cell extrusion using micropatterned surfaces

As both lamellipodia protrusions and actomyosin cables can exert mechanical forces^28,38,39^, we sought to understand how these forces contribute to successful extrusion. Therefore, we further dissected the mechanical work contribution by lamellipodia versus purse-string. From TFM measurements, our previous studies^28,38^ showed that during wound healing, lamellipodia exert traction forces pointing radially and away from the wound center, while purse-string traction forces are generally tangential or radially inwards. We thus adapted traction force microscopy to dissect the relative forces contributed to these two mechanisms during extrusion. We observed an increase of the overall traction forces upon cell extrusion as compared to forces at random sites (Supplementary Fig. 10a, b). By decomposing forces into radial and tangential components, we concluded that both components played a role in the force increase (Supplementary Fig. 10c), exhibiting an anisotropic distribution of forces at extrusion sites. Furthermore, the observation of inward- and outward-pointing forces with respect to the center of the extrusion zone suggested that both mechanisms, lamellipodia protrusion and purse-string, contributed significantly to cell extrusion, in agreement with wound healing and gap closure experiments^28,29^. As extrusion progressed, the purse-string mechanism appeared more pronounced (Fig.1a, Supplementary Fig. 10a) reflected by a more prominent contribution of inward-pointing forces (Supplementary Fig. 10a).

To further investigate the relative mechanical contributions of the two mechanisms at play during extrusion, we first measured the forces during extrusion over circular (10, 15, and 30-µm diameter) nonadhesive patches to determine the forces produced by purse-strings. The fact that contractile purse-string is partially connected to the focal adhesions at the edge of the patch indicates that the resulting forces generated by purse-string could be measured by traction force exerted on the edges of nonadhesive patches. Indeed, pronounced traction forces were observed at the edges of the nonadhesive patches during extrusion (Fig. 6d). These forces are prolonged for 2.5 h on patches of D = 30 µm and 15 µm corresponding to incomplete extrusion on these patches. On 10 µm diameter nonadhesive patches, the forces diminished after 60 min (the typical duration of extrusion). Also, on D = 10 µm the orientation of force was more anisotropic (Fig. 6e iii and Supplementary Movie 6) compared to prominent radial inwards-pointing forces observed on 15 µm and 30 µm diameter nonadhesive patches (Fig. 6e–i, ii, Radial Traction Force & Orientation of the forces with respect to extrusion center, Supplementary Movie 7). The dependence of the force pattern on the nonadhesive patch diameter corroborated with the reduced contribution of lamellipodia to extrusion as the patch size increased.

We estimated the tension contributed by purse-string using force measurement on nonadhesive patch of D = 30 µm. We represent the contractile force exerted by purse-string by a line under a tension denoted  τ (See Supplementary Note, Theoretical appendix). On nonadhesive patch of D = 30 µm, we observed that total and radial forces diminished together with the reopening of the closing area during extrusion (Fig. 6d–i, e–i, Supplementary Fig. 11a). Focusing on the nonadhesive case of radius R without lamellipodia protrusion, we observed conditions under which the closing area could reopened as determined by a zero velocity of the leading front (*dr/dt* = 0, Supplementary Fig. 11a). Under this condition, the mechanical equilibrium between the traction forces and the purse-string contraction allows estimating the line tension: *F*_*r*_ = *γ.2πR* where the radial force Fr = γ.2πR*Frr* (in N) is computed from the magnitude of the traction forces (in Pa) over a ring of radius R and width of 1 µm (See Theoretical appendix). From these measurements, we thus estimated that the line tension was *γ* = τ * R*^*−1*^ = 6.5 ± 1.4 nN µm^−1^ (a value consistent with the value reported in^40^ (Supplementary Fig. 11a, middle panel).

The estimation for purse-string tension was validated again on extrusion on nonadhesive patch size of D = 15 µm (Supplementary Fig. 11b). Note that at the range *γ* = 6.5 ± 1.4 nN *µ*m^−1^, the extruding area reduces linearly before reaching plateau. On patch size of D = 10 µm, we observed that the *F*_*r*_ only started increasing (Supplementary Fig. 11c – middle panel) at t = 40 min, corresponding to a radius around 2–2.5 µm. Since the nonadhesive patch remained smaller than the typical cell size, we expected that lamellipodia-based forces could contribute to cell extrusion over larger time scales than in the previous cases, leading to a delayed signal in solely purse-string based forces (Supplementary Fig. 11c).

**Fig. 7:**
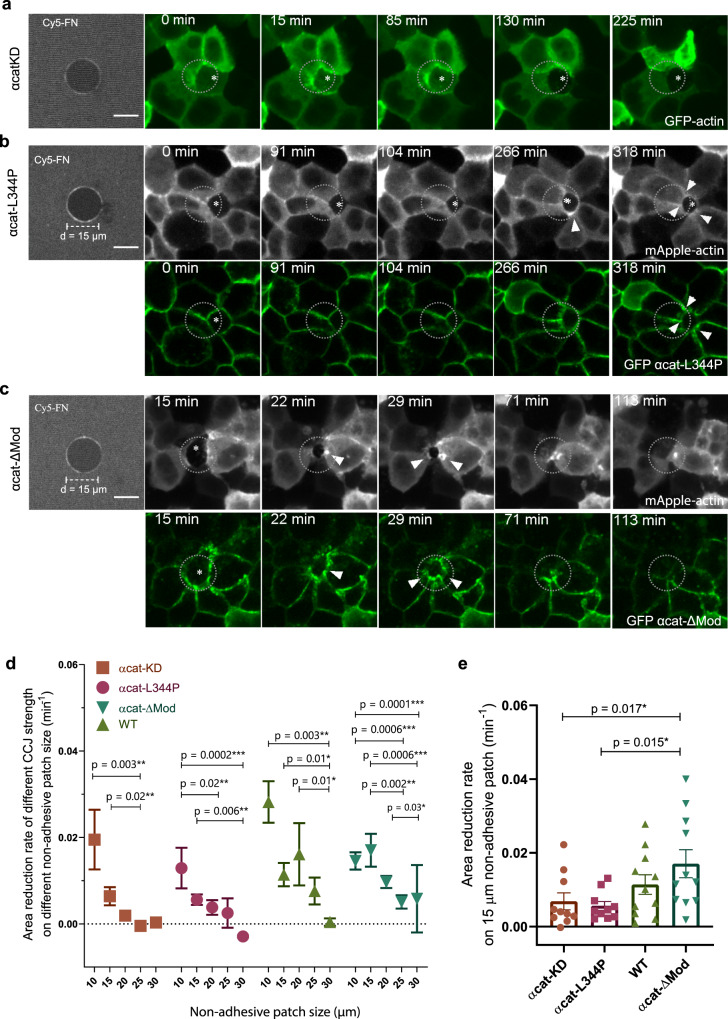
Interplay of cell-substrate adhesion and cell–cell junction strength on regulating dual-mechanism modes of extrusion. **a**–**c** Representative examples of extrusion of cells with different CCJ strength on 15 µm nonadhesive patches. The first image shows the substrate coated with Cy5 Fibronectin with darker field represents nonadhesive area. 2nd to 5th panel: confocal live imaging of **a** αcatKD cells **b** αcat-L344P cells, and **c** αcat- ΔMod cells. Arrowheads indicated the accumulation of αcat at tricellular points corresponding to cable formation. Scale bar = 10 µm. **d** Quantification of the mean area reduction rate of cell extrusion for the four types of cells on varying nonadhesive patch sizes. Error bars indicated SD. Nonparametric Kruskal–Wallis test was performed for each group (KD: *p* = 0.01*, L344P: *p* = 0.003**, WT: *p* = 0.037*, ΔMod: *p* = 0.0005***), followed by pair-wise comparisons (*p* values shown on graph). *N* = 10–28 for each condition. Source data are provided as a Source Data file. **e** Quantification of the rate of area closing for the same four types of cells on 15 µm nonadhesive patches. Error bars indicated SEM. Number of extrusion events: WT: *N* = 10; αcat-KD: *N* = 10; αcat-L344P: 3: *N* = 10; αcat-ΔMod: *N* = 11. At least two independent experiments were performed for each condition Nonparametric Kruskal–Wallis test (*p* = 0.04*) followed by pair-wise comparison was performed (*p* values shown on graph). Significant level: *p* < 0.05*; *p* < 0.01**. Source data are provided as a Source Data file.

We further investigated how cell–cell junctions strength modulated the forces contributed by purse-string during extrusion. We combined nonadhesive patches with the αcatKD, αcat-L344P, and αcat-ΔMod cell lines of varying CCJ strengths. We compared their behaviors on nonadhesive patches with diameters of 10, 15, 20, 25, and 30 µm with those of WT cells (Fig. 7). αcatKD cells failed to be extrude successfully from the nonadhesive patchs even at small sizes (Fig. 7a, Supplementary Movie 8). We also estimated the line tension produced by actin cables formed by αcatKD cells to be less than 1 *n*N *µ*m^−1^ (Supplementary Fig. 11d). Although there was actin accumulated around the extruding-neighboring interfaces and caspase-3 signal was elevated, the extruding area stagnated over 6 h. In αcat-L344P cells, the actomyosin cables were observed at CCJ as the extruding area reduced (Fig. 7b). However, most of the extrusions failed in this case because the actin cable was not sustained (Fig. 7b). On the other hands, extrusion of αcat-ΔMod cells was generally successful with visibly actomyosin cable reinforcement (Fig. 7c, white arrowheads). Furthermore, there were also successful extrusions for these cells on larger nonadhesive patches (Supplementary Movie 9). In summary, enhancing CCJ strength could increase the speed of closure on nonadhesive patches (Fig. 7d, e).

**Fig. 8:**
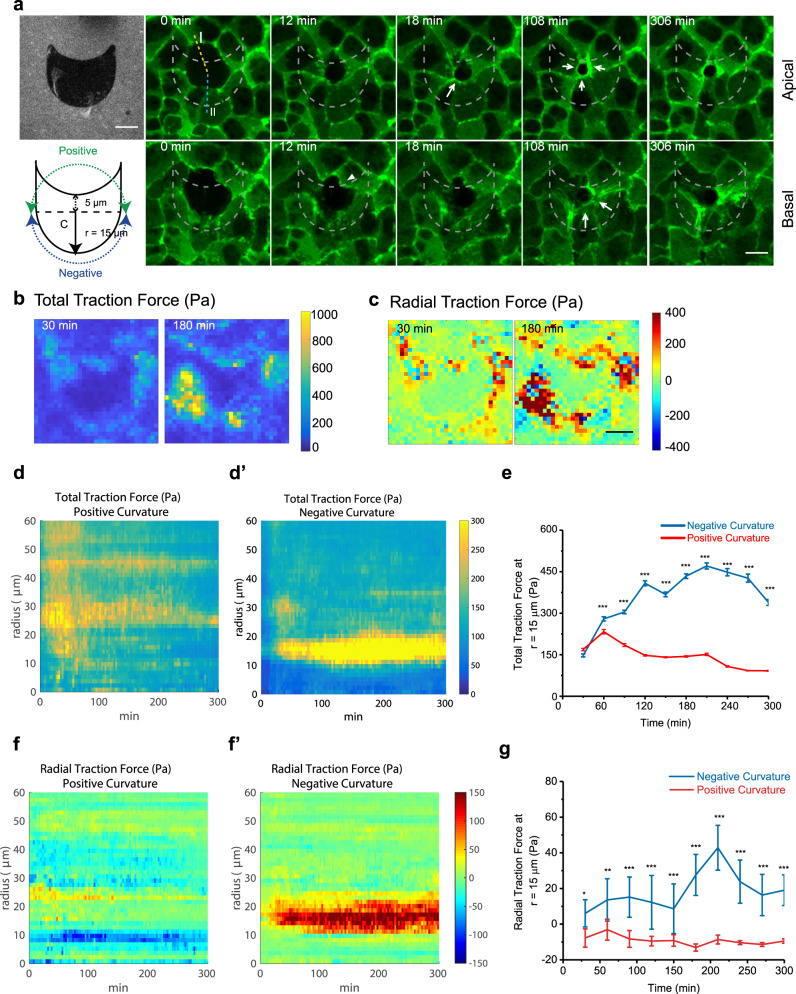
Anisotropic nonadhesive shape dictated preferential mode of extrusion. **a** Confocal time-lapse images of cell extrusion on nonisotropic nonadhesive shapes. First panel, first image: substrate coated with Cy5 fibronectin showing nonadherent patches (darker phase). Second panel, first image: schematic of the anisotropic patch design. 2nd–5th images: time-lapse confocal imaging at apical plane (top panel) and basal plane (bottom panel) of GFP-actin expressing monolayer centered on a cell extruding above a nonadherent patch (nonfluorescent cell). The negatively curved region induced the earlier formation of contractile actin cables (white filled arrow). Scale bar = 10 µm. **b**, **c** Average heatmap for total (**b**) and radial traction forces in Pascal (Pa) (**c**) averaged over four extrusion events (One event each experiment). The force magnitude was color-coded on the color scale bar (in Pa). Scale bar = 10 µm. **d–d’** Kymographs for total traction forces around extrusion sites for positively-curved (P) regions and negatively curved (N) regions. The force magnitude was color-coded on the color scale bar (in Pa). *m* = 4 extrusion events. **e** Averaged traction force magnitude at r = 15 µm (equivalent to the distance between center to negative edges) in function of time. *n* = 20 averaged force vectors for each time point, averaged from *m* = 4 extrusion events. Error bars indicate SEM. two-tailed unpaired *t*-test was used for comparing between negative curvature and positive curvature. In time series order: *p* values = 0.08, 7 × 10^−4^, 10^−12^, 10^−24^, 10^−22^, 10^−21^, 10^−20^, 10^−28^, 10^−28^, 10^−29^. **f–f’**. Kymographs for radial traction forces around extrusion sites for positively-curved (P) regions and negatively curved (N) regions. The force magnitude was color-coded on the color scale bar (in Pa). *n* = 20 averaged force vectors for each time point, averaged from *m* = 4 experiments. **g** Averaged radial traction force at r = 15 µm in function of time. Replicates number same as **e**. Error bars indicate SEM. Two-tailed unpaired *t*-test was used for comparison between negative curvature and positive curvature. In time series order: *p* values = 10^−6^, 10^−15^, 10^−19^, 10^−22^, 10^−23^, 10^−20^, 10^−19^, 10^−22^, 10^−20^, 10^−16^. Source data for **b**–**g** are provided as a Source Data file.

Finally, previous studies have shown that substrate curvature can promote either lamellipodia-based protrusions or actin cable assembly^29,38^. Along this line, we developed anisotropic patterns (Fig. 8) with a positive-curvature side, which could favor lamellipodia protrusion and a negative-curvature side, which could favor actin cable formation. Preferential formation of a cable at the negative curvature before the formation of actin cable on the positive curvature was observed (Fig. 8a). At the positive-curvature side, lamellipodia protrusion was dominant until the protruding front reached the edge of the patch and converted into actin cable (Fig. 8a, t = 12 min and t = 18 min, white arrowhead). The forces generated by lamellipodia protrusion and actomyosin cables were also dissected on these anisotropic nonadhesive shapes (Supplementary Fig. 12). As extrusion progresses, higher radial traction forces pointing towards the center accumulated at the negative edge than at the positive edges while the overall traction forces increased around the shape’s edges (Fig. 8b, c). On positive curvature, outward-pointing traction forces span the patch edge during early extrusion (Fig. 8d, f, Supplementary Fig. 12a, b, top panel, 0–30 min), that were attributed to the forward cell crawling movement. The force orientation became tangential to the edge of the cell as it crawled towards the edge of the nonadhesive patch (Supplementary Fig. 12a, b, top panel, 45 min). On the negatively curved region of the patch, forces oriented tangentially to the cell’s edge (Fig. 8d’, f’, Supplementary Fig. 12a’, b’, 45–72 min). Over the time, cells were pulled inside the nonadhesive area, leading to a change of force patterns with an increase of inward-pointing forces (Supplementary Fig. 12, 87 min, Fig. 8e–g). This could be explained by the formation of cables that linked cellular front over the nonadhesive area to remaining adhesions at the back (Fig.4 8a, 108 min). Altogether, these data helped to clarify the force pattern and the mechanical signature of the transition associated to lamellipodia and actin cable assembly.

### A theoretical model to reconcile forces produced by purse-string and lamellipodia protrusion during extrusion

**Fig 9:**
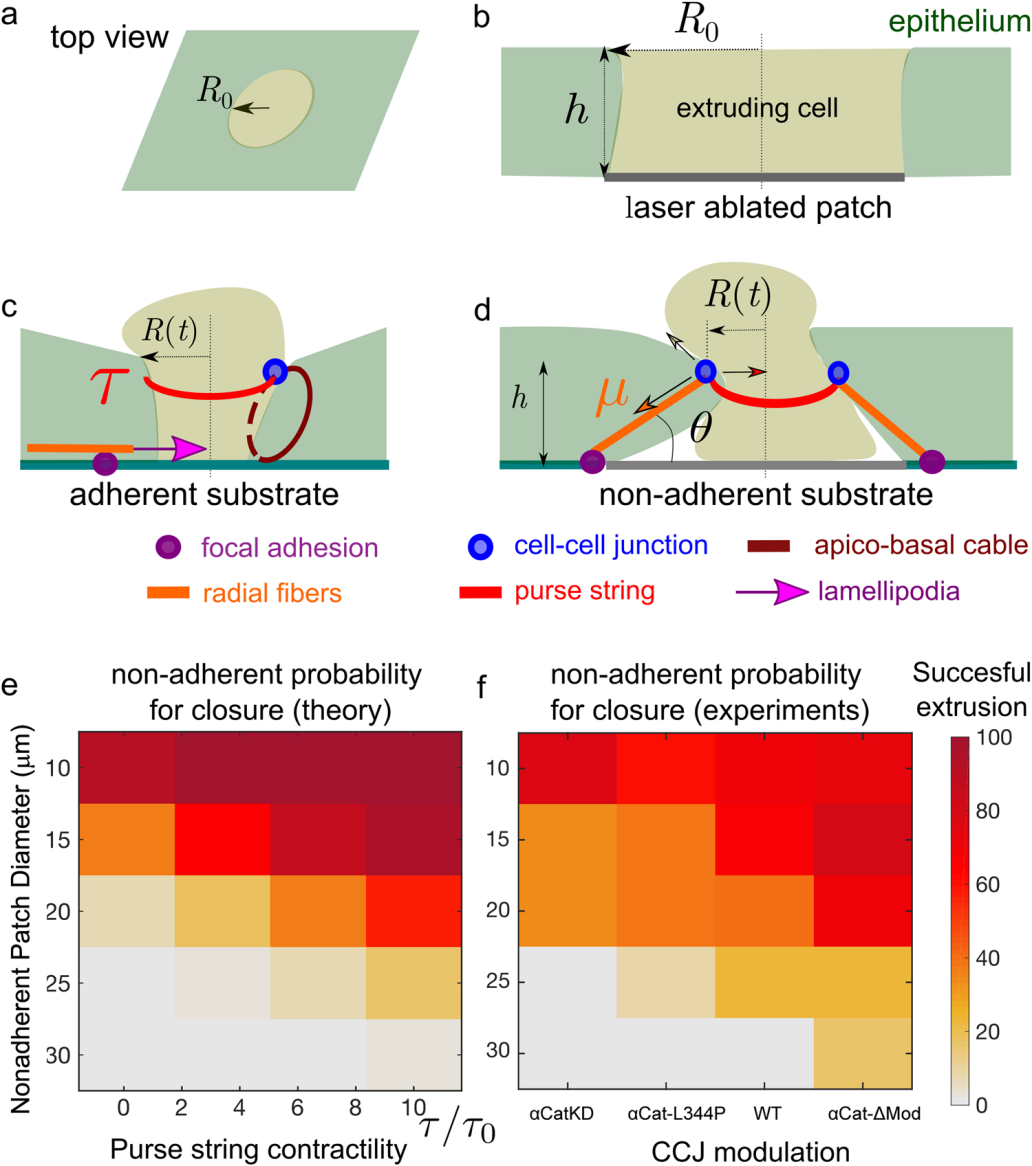
Integrative model for extrusion. **a**–**d** Sketch of the model. **a** Side view with the extruding cell (represented as a light green disk) and the surrounding epithelium. **b** Side view at the initial stage of extrusion with the laser ablated patch (corresponding to one or several cells) of a radius R0. **c** Adherent case: a combination of lamellipodia, apico-basal cable, and nonuniform purse-string contribute to the extrusion process. **d** Nonadhesive case: a multicellular purse-string of tension τ (red circle) contributes to the extrusion dynamics while apico-basal cables (orange line), with a tension denoted μ, provide a resistive contribution. Arrows represent the forces acting on a cell–cell junction (represented by a blue circle). **e** Phase diagram of the predicted percentage of successful extrusions in terms of the nonadhesive patch diameter (2 *R*_*0*_) and of the purse-string contractility. **f** Phase diagram of the experimentally measured percentage of successful extrusions in terms of the nonadhesive patch diameter and of the CCJ perturbation. Number of extrusion events: N = 10–28 for each condition. Source data for **e**, **f** are provided as a Source Data file.

We developed a theoretical model that provides an integrated view of the extrusion mechanism driven by cell protrusions and cellular contractility. In two recent works, a theoretical framework emerged to account for the high variability of the healing time in wounded tissues^40,41^. In spite of similarities, including in terms of time scales, the length scales involved in extrusion are significantly lower than in the wound closure experiments reported in refs. ^40,41^, indicating discrepancies in the strength of the forces involved in these processes. Our theoretical model reconciled the relevant forces involved in extrusion and wound closure, as well as the two experimental features observed here in the context of extrusion over adhesive and nonadhesive substrates (see Figs. 9a–d).

Our model is based on a description of the bulk tissue as a continuum, incompressible viscous material characterized by a homogeneous and isotropic viscous modulus ( η). Such description is motivated by the large duration of the extrusion process compared to previous visco-elastic time estimates (1 h timescale in ref. ^42^).

We express the stress exerted at the boundary of the extruding cell as

1 $$\sigma _{r = R\left( t \right)} = P_c + \sigma _P + \sigma _\mu + \frac{\tau }{{R(t)}},$$

(see Supplementary, Theoretical Appendix, Eq. 4) where *R*(t) is the radius of the closing area; *P*_*C*_ is the initial monolayer prestress at t = 0 of ablation; *σ*_*P*_ > 0 corresponds to the protrusive stress exerted at the interface of the neighboring cells^40,41^); *τ* is the tension along the actomyosin cables within the multicellular purse-string located on the apical side; finally, we introduce a term *σ*_*μ*_ to account for the stress exerted through radial actin cables, providing a resistive contribution to extrusion (*σ*_*μ*_ < 0) in the case of nonadhesive patches. We then found that the rate of extrusion can be expressed as:

2 $$\left( {\xi R\,{\mathrm{Log}}\left[ {\frac{{R_{{\mathrm{max}}}}}{R}} \right] + 2\,\eta } \right)\dot R = - P_c - \sigma _P - \sigma _\mu - \frac{\tau }{{R(t)}},$$

where *ξ* is a substrate friction coefficient; *η* is a shear viscosity and *R*_max_ ≈ 100 µm a hydrodynamic screening length (see Supplementary Information). Solving the equations for *R*(*t*) as the function of time (Eq. 2), we found that the completion probabilities and timing for the adhesive substrate is largely independent of the value of the contractility by the actomyosin cable. This is consistent with our experimental results that modulating actomyosin cables contraction via changing CCJ strength does not significantly alter the duration of extrusion (Fig. 5f).

The difference in the nonadhesive case lies in the existence of radial stress fibers that spread from the edges of the nonadhesive patch (basal side) to the edge of the tissue (apical side, see Fig. 6b, Fig. 9c, d, Supplementary Fig. 8d). As they were shown to be tensile (Supplementary Fig. 8a–d’), these radial cables resist the closure activity of neighbors. These fibers become increasingly tilted in the apico-basal direction (Fig. 9d, Supplementary Fig. 8d’), and these angles are correlative to the strain exerted when closure occurred on nonadhesive patches (Supplementary Fig. 8e). Taking into these observations, we consider the simplest assumption of constant contractility *μ* along these fibers. In turn, the projected in-plane tissue stress increases during the extrusion process due to the increased projection of forces with the increasing tilt of the fibers with the substrate (see Supplementary Note—Supplementary Fig. 14). We experimentally showed that the cell extrusion failed whenever the size of the nonadhesive patch is larger than a critical radius that can be estimated in our model (see Supplementary Note—Supplementary Fig. 15).

Estimates for the absolute value of the pressure field are expected to be in the Pc = 10–100 Pa. µm = 0.01–0.1 nN. µm^−1^ range^43^. The corresponding forces appear small (overall in the 1 nN) compared to the forces associated to lamellipodia or purse-string structures, whose strength can be estimated in the 10 nN range based on the value of traction forces close to the extrusion site (see Fig. 6d).

Being irreversible, stochastic fluctuations could drive the extrusion to completion even when average forces would prevent it. By adding a mechanical stress fluctuation term *ξ* into Eq. 1, we show that fluctuations can crucially contribute to the statistics of the closure of the extrusion site (see also Supplementary Note—Eqs. 10 and 11). We used numerical simulation to solve the probability of extrusion as the function of initial size and probability according to this framework. We compared our simulations with experiments performed with cells expressing different αcat mutants (Fig. 9e, f) on different nonadhesive patch sizes. The trend obtained from numerical solutions varying the strength of the purse-string contractility (see Fig. 9e, f) closely follows the trend observed in experiments with different αcat mutant background for cells on nonadhesive patches. Our analytical model demonstrated that the presence of fluctuations may lead to the completion of the extrusion process even in a phase space of parameters where deterministic forces would prevent it from occurring. We estimated the level of fluctuations from the autocorrelation of the temporal fluctuations in the averaged radial force. We found that stress fluctuations were higher for α-catenin KO than for wild-types (see Supplementary Fig 13a–c); incorporating an increased level of fluctuations of αcat mutants in our stochastic simulation still lead similar extrusion probability as in Fig. 6 (see Supplementary Fig 13d–f).

The assumption of a viscous bulk tissue undergoing fluid flows is not incompatible with the assumption of a constant prestress exerted by the apico-basal cables. In spite of an expected fast turnover time of focal adhesion (in the min timescale), new focal adhesions are constantly formed within a specific region at the edges of the nonadhering substrate; similarly, in spite of an expected fast remodeling time of individual actin fibers (in the min timescale), the apico-basal fibers can remain stable (e.g., with stable anchoring over the substrate) over the whole duration of the experiments.

## Discussion

Our experimental and theoretical findings establish a quantitative scenario for the mechanisms driving cell extrusion in an epithelial sheet. It provides a holistic model of the joint efforts of two distinct cellular processes, lamellipodia protrusions and contractile actomyosin cables to eliminate apoptotic cells from tissues while maintaining epithelial integrity. Branched actin networks of lamellipodia provide fast pushing forces on the membrane to close the extruding area early at the basal plane. Contractile actomyosin cables first form at apical planes in a discontinuous manner and only become uniform at later stages of extrusion. Lamellipodia assembly leads to protrusive activity underneath the dying cell but also helps to anchor actomyosin cables in apical-to-basal direction. Hence, the tilted localization of the complete purse-string rings shows unanticipated closure processes, which are following not only horizontal but also apico-basal directions.

The dual-mechanism mode involving lamellipodia protrusion and heterogeneous purse-string could be adjusted by changing cell-substrate adhesion patterns or AJ strength. Such dual-mechanism ensures that basal sealing is accomplished and further maintains epithelial integrity during apoptotic extrusion. Our results provide additional models besides the ones already published^3,19,44^, which emphasized the dominant role of a complete multicellular purse-string that fully surrounds the apoptotic cell during extrusion.

The combined effect of lamellipodial protrusions and actomyosin contractility during extrusion is well captured by the observed force patterns and our theoretical approach. We delineated the efficient forces exerted by the purse-string or lamellipodial protrusions that should overcome the bulk resistance of the epithelial monolayer by both experimental traction force analysis and the analytical model. We were able to deconstruct experimentally the force values for lamellipodia and purse-string during extrusion. The purse-string formation results in forces pointing radially towards the center of extruding cell on top of nonadhesive patches as extrusion progresses with a measured line tension of 10–13 nN. These force magnitudes corroborate with those measured on large wound closure^28,45^. The estimated stress exerted by lamellipodial protrusions was between 50–100 Pa, in agreement with previous measurements^10,46^. By theoretical modeling and comparing the numerical solution with experimental data, we unveiled the relative contributions of forces at play to expel dying cells from epithelial monolayers.

Heterogeneous mechanisms have been proposed to facilitate wound healing and gap closure^28,29,47,48^. These studies emphasized that the full, uniform purse-string was important yet sometimes not sufficient to promote gap closure. Recent in silico studies suggested that a mixed mechanism of contractile purse-string and protrusive crawling were more efficient than single-mechanism-based closure^49,50^. Similarly, experiments done in this work prove that a dual-mechanism is essential for cell extrusion. We provide further information on how lamellipodia-driven basal protrusions could affect subsequent purse-string formation three dimensionally. This is strikingly different from the case of wound closure since, in extrusion, the closing activity is coupled with partial apico-basal changes when the extruding cell gradually loses contact with its neighbors. In wound healing, actin reorganization at the front of the wound only occurs in two dimensions.

The heterogeneous changes of actin structures around the extrusion site were found to be coupled with the nonuniform remodeling of CCJ between the extruding cell and its neighbors. Previous studies showed that the reduction of E-cadherin at the junctions, together with a reduction of tension at the lateral membrane, occurs before extrusion^20,24,51^. The reduced tension at the CCJs is necessary for increased contractility that expels the cell^24^, and redistribution of E-cadherin clusters is crucial to recruit factors organizing actomyosin purse-string like Coronin 1B^27^. Here we provide an additional role of AJ in mediating extrusion via dual-mechanism mode. The stochastic formation of lamellipodia at early extrusion stages led to an asymmetrical partial loss of apico-basal polarity and reduction of AJ at each neighbor-extruding cell interfaces, which corresponds to inhomogeneous actomyosin purse-string formation. Furthermore, while cadherin-catenin complexes are reduced at bicellular interfaces, they appear to be enhanced at tricellular contacts together with the recruitment of actomyosin cables, suggesting that there is likely positive feedback between AJ reinforcement and actomyosin cable maintenance.

Taken together, we established the interplay of the cell–cell junction and cell-substrate in regulating the extrusion mechanism by tuning lamellipodia protrusion and contractile purse-string mode. The switching mechanism from lamellipodium protrusion to contractile purse-string helps epithelia adapt to the environment with heterogeneous substrate adhesion molecules, which typically occurs in vivo to maintain homeostasis. The complex and heterogeneous regulation, even at a small-scale event, epitomizes the robustness of epithelia and proposes a framework for understanding epithelial homeostasis as well as extrusion-related pathological conditions.

## Methods

### Cell line and tissue culture

Madin-Darby canine kidney (MDCK) strain II was cultured in DMEM medium (Invitrogen) supplemented with 10% FBS (Invitrogen). To study the function of specific proteins, stable cell lines or transient expression/knockdown variant of MDCK were used. Stably transfected cells including GFP-actin MDCK, Lifeact Ruby MDCK, α-catenin knockdown MDCK, mCherry E-cadherin MDCK, and GFP-E-cadherin MDCK are kind gifts from James W. Nelson. MDCK PBD-YFP expressing MDCK was kindly provided by Fernando Martin-Belmonte, Universidad Autónoma de Madrid. These stably transfected cells are maintained with media supplemented with 250 μg/mL geneticin (Invitrogen) to maintain their gene expression. Fluorescence-labeled cells are checked regularly for uniform fluorescence intensity and selected by cell sorting if necessary. Mycoplasma test was done every three months to control the mycoplasma contamination.

### Plasmids and transfection

To study the role of CCJ during extrusion, we manipulated α-catenin expression. The α-catenin mutants were published previously^35^: L344P mutant with the leucine to proline point mutation at the 344aa of VH2 domain conferring α-catenin’s inability to bind to vinculin, and Δ-Mod mutant which has the vinculin-binding modulatory domain deleted (aa 396–631) and allows α-catenin to be constitutively bound to vinculin. These mutant constructs were transiently transfected in α-catenin knockdown MDCK cells to generate cell populations with varied CCJ strength. In this study we used: α-catenin knockdown (αcatKD), α-catenin-L344P (L344P), wild-type α-catenin (WTres), α-catenin Δ-Mod (ΔMod). mEmerald-Myosin-IIA-N, mApple-Actin, GFP-Actin, mCherry-ARPp34-N were kindly shared by Pakorn Kanchanawong. GFP-APHP RhoA sensor was the generous gift from Alpha Yap. Transfection was performed with Neon electroporation system (Invitrogen).

### Laser induction system to study apoptotic extrusion

We induced apoptosis in monolayer by UV laser to be able to capture early events of extrusion. The system was designed as in ref. ^52^, consisting of a Nikon A1R MP laser scanning confocal microscope, with Nikon Apo 60 X oil-immersion/1.40 objective. To induce apoptosis, UV-laser (355 nm, 300 ps pulse duration, 1 kHz repetition rate, PowerChip PNV-0150-100, Teem Photonics) was focused at the target cell for 7–10 s with the power of 10 nW at the back aperture. With this setting, DNA-strand break is induced without permeabilization of the membrane^10^. This induces cell undergoing apoptosis and being extruded.

### Cell seeding setup

To distinguish lamellipodial protrusion and actin cable formation at the extruding cell-neighboring cells interface, we used mosaic cocultures of nonfluorescent cells (which would be laser-induced for apoptosis) and fluorescence-labeled GFP-actin or Lifeact Ruby cells at the ratio of 1:7. Cells were trypsinized, mixed and seeded at 2 × 10^6^ cells/µm^2^ on glass bottom petri dish (Iwaki) coated with fibronectin (Roche, 1 h incubation, 25 µg/mL) or on fibronectin-micropatterned substrates. Sixteen to twenty hours before imaging. Cell attachmente was monitored every half an hour and unattached cells were washed by PBS. Cells density was kept at the range of 40–45 cells per 100 × 100 µm^2^ unless otherwise specified for density-dependent measurements.

### Determination of extrusion initiation (t = 0 min)

Initiation of apoptotic extrusion was defined by (i) the brightening of caspase-3 indicator, (ii) a bright spot on phase contrast or bright field image, (iii) the sharp shrinkage of the cell area, and (iv) the initiation of nucleus condensation and fragmentation. Our previous published results indicate that these four hallmarks occur simultaneously within the time interval of interest (3–5 min) followinglaser induction pulse^10^.

### Drug treatment

Drugs were incubated 2 h prior to imaging unless stated otherwise. The drug concentrations are as follow: (S)-nitro-blebbistatin (Cayman Chemical) 50 μM, NSC-23766 (Sigma–Aldrich) 200 µM, SMIFH2 (Sigma) 50 μM dissolved in DMSO. Caspase-3 indicator DEVD-FMK conjugated to Sulfo-rhodamine (Abcam) was used at 1:1000 dilution and incubated with the cells 30 min before experiment. To visualize F-actin in certain experiments, siR-actin (Cytoskeleton) was added at 100 nM concentration to the medium 12 h before imaging. Hoechst 33342 at 1 µg/mL (Sigma) was added into culture media 1 h and washed before imaging for nucleus live detection.

### Micropatterning of the substrate

Wafers with the custom-designed patterns and micropillars were prepared following photolithographic techniques as described previously^53^.

For cell monolayer confined on patterns, a stenciling technique was used. PDMS (Sylgard 184, Dow Corning) was mixed with crosslinking agent to the ratio of 10:1. The mixture was poured onto the wafer and degassed then cured at 80 °C for 2 h. The stencil with patterns of interest was deposited onto the glass-bottomed IWAKI dishes and plasma-treated with Oxygen for 30 min. By this way, the nonpatterned area on glass surface became functionalized and could attach to 0.1 mg/mL PLL-g-PEG (SuSoS) that was flowed in later by capillary. The dish was incubated with PLL-g-PEG for 1 h before removing the stencil and coated with fibronectin. Fibronectin was washed off and the surface was further treated by 2% pluronics (F127, Sigma) for further passivation of nonpatterned area.

For experiments with nonadhesive patches, microcontact printing was used instead of microstenciling. PDMS substrate was spin-coated at 1000 rpm for 30 s then 4000 rpm for 30 s on IWAKI petri dishes and cured for 1.5 h at 80 °C. PDMS stamps were prepared from wafer molds and coated with mixture of 50 μg/mL fibronectin (Roche) and 25 μg/mL Atto647-conjugated fibronectin (Atto dye, Sigma). Stamps were deposited on deep UV-treated surfaces (15 min) and the unstamped areas were passivated by 2% pluronics (F127, Sigma) for 2 h to render nonadhesive surface. The dishes were rinsed with PBS three times before seeding cells.

### Immunostaining and antibodies

Cells were fixed with 4% PFA at 37 °C for 10 min, permeabilized and blocked with 0.1% Triton X-100 in 1% BSA/PBS overnight. Myosin-IIA was stained using rabbit Anti-myosin-IIA antibodies (Sigma M8064) 1:100. Paxillin was stained using rabbit monoclonal anti-paxillin antibodies [Y113] (Abcam ab32084) 1:100. Actin filaments were stained with Alexa Fluor® 568 Phalloidin (Life Technologies, A12380) 1:100, Alexa Fluor® 488 Phalloidin (Life Technologies, A12379) 1:100 or Alexa Fluor® 647 Phalloidin (Life Technologies, A22287) 1:100. Secondary antibodies Goat Anti-rabbit IgG Alexa Fluor® 568 (Life Technologies, A-11011) were used at a 1:100 dilution. Nucleus was labeled with Hoechst 33342 at 1 µg/mL concentration.

### Microscopy

Time-lapse confocal imaging was carried out with spinning disk confocal microscopy (Nikon Eclipse Ti-E inverted microscope body, CSU-W1 Yokogawa head, dichroic filters and 1.27 NA ×60 water-immersed objective). GFP fluorescent signal was taken with 488 nm Diode laser, 50 mW, 5% power, 100 ms exposure. RFP/mCherry/mApple fluorescent signal was taken with 561 DPSS laser, 25 mW, 5% power, 100 ms exposure. Images were taken with z-step = 0.5 µm, 17–19 stacks. Super-resolution imaging was done using the Live-SR module (York et al., 2013) integrated with the above-mentioned spinning disk confocal microscope using a 1.6 NA ×100 oil-immersed objective. GFP fluorescent channel was taken with 488 nm Diode laser, 50 mW, 8% power, 100 ms exposure. RFP/mCherry/mApple fluorescent signal was taken with 561 DPSS laser, 25 mW, 10% power, 100 ms exposure. DAPI fluorescent channel was acquired with 405 nm Diode laser, 50 mW, 5% power, 100 ms exposure. Images were taken with z-step = 0.1 µm. Microscope settings were kept constant throughout the set of experiments for keeping consistent quantification.

### Traction force microscopy

For traction force microscopy, a substrate of 10-20 kPa Young’s Modulus was prepared by mixing CyA and CyB PDMS components (Dow Corning) at the ratio of 1:1 to enable detection of nanoNewton forces. The mixed PDMS was spin-coated onto glass-bottomed petri dishes (IWAKI) and cured in 80 °C for 30 min. The surfaces were then incubated with 5% (3-Aminopropyl)trimethoxysilane (Sigma) in ethanol for 5 min. carboxylated red fluorescent beads (100 nm, Invitrogen) were then functionalized on the substrate at a 1:500 dilution in deionized (DI) water. The beads were passivated with 100 mM Tris solution (Sigma) in DI water at for 10 min. Finally, fibronectin (50 µg/ml) was incubated for 1 h at 37 °C. This substrate was patterned on soft gel using indirect microcontact printing. First, the pattern of interest was stamped first on thin Polyvinyl Alcohol membrane made up of 5% PVA solution (Sigma) and transferred the inverted membrane to the substrate. The membrane was dissolved, and the nonpatterned areas were passivated by incubation with 2% Pluronics for 2 h. Finally, cells can be seeded onto the substrate as previously described.

The bead displacement was calculated using PIVlab (Thielicke & Stamhuis, 2014). The settings are: (1) Fast-Fourier Transform (FFT), (2) a Gause 2-by-3 point subpixel estimator, (3) linear window interpolator, and (4) three-passes (64 × 64, 32 × 32, 16 × 16 pixel size interrogation window equivalent to 12 × 12, 6 × 6, 3 × 3 µm^2^ with 50% overlap,). Bead displacements were converted into traction force (in Pa) by Fourier Transform method using FTTC ImageJ plugin developed by Martiel et al. (2015). The subsequent analysis was done on customed-written Matlab scripts.

### Laser ablation

The laser ablation system composed of a UV-laser (355 nm, 300 ps pulse duration, 1 kHz repetition rate, PowerChip PNV-0150-100, team photonics) was integrated into a Nikon A1R MP confocal microscope, with customized optical path and dichroic filter coaligned with the optical axis of the microscope. Position of the laser can be controlled by a mirror mounted on two linear actuators (TRA12CC, Newport), and the exposure time of the laser was controlled by a mechanical shutter (VS25S2ZM0, Uniblitz) using built-in ImageJ plugins from a PC. The system was mounted on the spinning disk confocal system described above equipped with ×60 oil-immersed/1.4 NA objective. The integrated laser ablation system allows us to perform laser ablation during imaging.

Laser ablation was performed on cells expressing fluorescent-labeled myosin regulatory light chain (MRLC) to visualize actin cables Apoptosis was induced as previously described and actin cable formation monitored by imaging at time interval of 10 min. Once the cables were visualized, a time-lapse image series was launched on the region of interest (256 × 256 pixels) at time interval of 1–2 s. A UV-laser pulse of 5nW, 5 s was performed on the desired myosin-rich region. Images were then taken for 5 min. The recoil distance of the actin cable d(t) was determined by tracking the displacement of the two ends connecting to cell–cell junctions. The cable could be modeled as a Kelvin-Voigt fiber (Fernandez-Gonzalez et al., 2009) by fitting into equation: $d\left( t \right) - d\left( 0 \right) = \frac{{F_0}}{E} \times \left( {1 - e^{ - \left[ {\left( {\frac{E}{\mu }} \right) \times t} \right]}} \right)$

*d(t)* as the recoil distance at time t

*d(0)* as the original distance between two CCJ.

*F0* is the tensile force at the junction before ablation

*E* is the junction’s elasticity

*μ* is the viscosity coefficient related to the viscous drag of the cell cytoplasm

The initial recoil, as such, could be derived into equation: ${\mathrm{Initial}}\,{\mathrm{recoil}} = \frac{{F_0}}{\mu }$

By fitting each ablation event into the equation, we could derive the initial recoil value, which is proportional to the tension exerted at the junction by actin cable.

### Image analysis

In order to obtain statistics, image acquisition settings of the same type of experiments were kept constant. Images were background subtracted and contrast enhanced for visualization. Intensity measurements were performed on raw images. Fluorescence intensities at the extruding cell-neighboring cell edges were measured using line scan tool in ImageJ. Bleach correction for confocal time-lapse imaging was performed using ImageJ EMBL Bleach Correction plugin. Afterwards, we took the summation image for stack of six slices (z-step = 0.5 μm, from 1 to 3.5 μm above the basal plane). Intensity profiles of actin or myosin at cell–cell junctions were obtained by performing a 10-pixel line scan spanning along the junction and corrected by deducting the average fluorescence intensity of cell contributing to the junction of interest. Average intensity and standard deviation values were obtained from this final line profile. Inhomogeneity of the actin/myosin intensity was defined as Inhomogeneity = Mean Intensity/90th percentile Intensity of the line profile, or as standard deviation normalized by the mean intensity. To correlate the formation of actin cables with the stage of extrusion, we used caspase-3 indicator DEVD-FMK conjugated to Sulfo-rhodamine (Abcam) as the surrogate marker for the extrusion stage.

To measure the actomyosin ring localization in 3D, we measured myosin-IIA intensity on fixed cells imaged by Structured Illumination Microscopy (Nikon Eclipse Ti-E inverted microscope body, CSU-W1 Yokogawa head, dichroic filters. The 3D reconstruction was performed on Imaris, and the ring localization can be tracked using manual point selection tool to select the myosin intensity at cell–cell interface. The coordinations of points corresponding to the actomyosin rings were fitted into a plane by matlab. By calculating the angle between this plane and horizontal plane, the angle that the ring forms with horizontal plane can be deduced.

### Reporting summary

Further information on research design is available in the Nature Research Reporting Summary linked to this article.

## Supplementary information

Supplementary Information

Peer Review File

Descriptions of Additional Supplementary Files

Supplementary Movie 1

Supplementary Movie 2

Supplementary Movie 3

Supplementary Movie 4

Supplementary Movie 5

Supplementary Movie 6

Supplementary Movie 7

Supplementary Movie 8

Supplementary Movie 9

Reporting Summary

## Data Availability

The data supporting the findings of this study are available within the article and Supplementary Information or from the corresponding author upon reasonable request. A reporting summary for this article is available as a Supplementary Information file. Source data are provided with this paper.

## Source data

Source Data

## Code availability

The custom codes for data visualization and plotting of traction force microscopy on MATLAB are deposited on Github (https://github.com/leanhphuong201/extrusionnatcomm).
