# Supplementary material for: Adhesion-mediated heterogeneous actin organization governs apoptotic cell extrusion
Source: Nat Commun. 2021 Jan 15;12:397. doi: 10.1038/s41467-020-20563-9 (PMC7810754; doi:10.1038/s41467-020-20563-9)
Supplement: Supplementary file 3 — Descriptions of Additional Supplementary Files [file 41467_2020_20563_MOESM3_ESM.pdf]

Descriptions of Additional Supplementary Files

Supplementary Movie 1

Description: Example of cell extrusion with basal lamellipodia protrusion and inhomogeneous apical actin cables each files as Supplementary Movie/Audio/Data 1, etc. formation as the main mechanism. MDCK LifeAct Ruby (fluorescent label for F-actin) (middle) undergoes extrusion from monolayer. Basal plane (magenta) is superimposed with the apical plane (green, 4 μm above the basal plane). Neighboring cells can form either lamellipodia protrusion (closed arrows) or actin cables (open arrows) at individual cell-cell contact and in a heterogeneous manner during extrusion.

Supplementary Movie 2

Description: Lateral-basal protrusion affects apical cable heterogeneity. MDCK stably-expressing GFP-tagged Myosin Regulatory Light Chain (MRLC) and transfected with TdTomatoFTractin to distinguish contractile actomyosin cables with actin accumulating at protrusions. The first two panels show slice at +2 μm. The last panel showed maximal projection of the GFP-MRLC channel with z-color coded. Stack size = 0.5 μm. Note that the myosin at apical sides started to be inhomogeneous. At late extrusion, a uniform ring was first seen at the most apical side (72 min) but became inhomogeneous. The fluctuating membrane protrusion at lateral planes (after basal closures) precedes myosin accumulation at lower planes, suggesting that the apical actomyosin ring was pulled down by polymerization at more basal sides. This results in the partitioning of the ring into multiple ring structures that finish the process.

Supplementary Movie 3

Description: Cell extrusions in α-catenin-knockdown rescued with different α-catenin. Examples of confocal time-lapse images showing cell extrusion (asterisks) in scenarios of α-catenin rescue. mApple-actin was co-expressed as the marker for actin structures. The transfection of different α-catenin mutants into αcatKD cells rescued the contribution of actomyosin cables with different degrees of preference. Apical plane images were the average of signals from +2.5-5 μm above the basal plane. Lamellipodia protrusion is indicated by arrowheads, and apical actin cables are indicated by open arrows.

Supplementary Movie 4

Description: Extrusion of cells sitting on top of the non-adhesive patch. MDCK cells stably expressing mCherry-Ecadherin (WT) were transfected with GFP-actin. Confocal images show maximal intensity projection from the basal plane to + 2μm. The cell in the middle of the patch (D = 30 μm) was induced by laser for apoptosis. Note that there are three major events: i) Isotropic cable formation (at 1:03 hour). ii) Dissociation of E-cadherin from the extruding cell-neighboring cell contact (at 1:15 hour) and neighboring cells lose apico-basal polarity followed by iii) Enhancement of Ecadherin tricellular contact with more visible isotropic cable (open arrows, at 1:48 hour onwards). The dying cell, although released itself from the neighbors, was not extruded from the monolayer, and the gap created was not being sealed after 6 hours post-apoptosis.

Supplementary Movie 5

Description: Laser ablation on radial cables connecting to purse-string ring. Another representative of Supplementary figure 8 experiment. Laser ablation was performed on the radial actin fiber connecting to purse-string ring (actin labeled with GFP tag, mCherry-E-cadherin stably-expressed MDCK cells).

Supplementary Movie 6

Description: Traction force for cell extrusion on nonadhesive patch with D = 10 µm. Confocal images of cells labeled with GFP-actin superimposed with traction force map. The forces are color-coded according to the cosine of angles θ at which the traction forces formed with respect to the center of the patch were color-coded. +1 indicates forces pointing towards the center of the extruding cell (inwards), and -1 indicates forces pointing away from the center of extruding cell (outwards). Note that increased traction forces were followed extrusion at was correlative to the actin accumulation (indicating apical actin cables), which are inhomogeneous. Outwards forces (lower right-hand-side) were associated with the cells exhibiting crawling behaviors (which show visible edge movement before actin is prominent).

Supplementary Movie 7

Description: Traction force for cell extrusion on nonadhesive patch with D = 15 µm. Confocal images of cells labeled with GFP-actin superimposed with traction force map. The forces are color-coded according to the cosine of angles θ at which the traction forces formed with respect to the center of the patch were color-coded. +1 indicates forces pointing towards the center of the extruding cell (inwards), and -1 indicates forces pointing away from the center of extruding cell (outwards). Note that forces are pointing inwards, corresponding to uniform actin accumulation, and increased in size as extrusion progresses.

Supplementary Movie 8

Description: α-catenin-knockdown cell is unable to extrude on top of small non-adhesive patch with D = 15 µm. Example of α-catenin knockdown cells transfected with GFP-actin to visualize actin dynamics undergoing extrusion on top of the small nonadhesive patch (the size at which WT cells can be typically extruded). Images show averaged signals from +2.5-5 µm above the basal plane. Note that even though the cells undergo apoptosis with caspase-3 indicator signal turned on and actin accumulation at cell-cell interface, the cells were unable to be expelled from monolayer even after 6 hours.

Supplementary Movie 9

Description: Enhanced CCJ strength can help cells being extruded on large-size non-adhesive patches. Example of αcatenin ΔMod rescued cells undergoing extrusion (asterisk) on top of a large non-adhesive patch (D = 25 µm), the size at which weaker CCJ cells are typically failed. Images show maximal projection signal from the basal to +6µm to visualize CCJ throughout the process, when the neighboring cells lose apico-basal polarity. Note that neighboring cells can form actin cables (indicated by visible actin recruitment to the CCJ and enhanced CCJ at tricellular contact, closed arrowheads on the images).
