# Supplementary material for: Adhesion-mediated heterogeneous actin organization governs apoptotic cell extrusion
Source: Nat Commun. 2021 Jan 15;12:397. doi: 10.1038/s41467-020-20563-9 (PMC7810754; doi:10.1038/s41467-020-20563-9)
Supplement: Supplementary file 13 — Reporting Summary [file 41467_2020_20563_MOESM13_ESM.pdf]

nature research

Corresponding author(s): Jean-François Rupprecht, Chwee Teck Lim, Benoit Ladoux

Last updated by author(s): Nov 18, 2020

Reporting Summary

Nature Research wishes to improve the reproducibility of the work that we publish. This form provides structure for consistency and transparency in reporting. For further information on Nature Research policies, see Authors & Referees and the Editorial Policy Checklist .

Statistics

For all statistical analyses, confirm that the following items are present in the figure legend, table legend, main text, or Methods section.

n/a	Confirmed	
☐	☒	The exact sample size (*n*) for each experimental group/condition, given as a discrete number and unit of measurement
☐	☒	A statement on whether measurements were taken from distinct samples or whether the same sample was measured repeatedly
☐	☒	The statistical test(s) used AND whether they are one- or two-sided<br>*Only common tests should be described solely by name; describe more complex techniques in the Methods section.*
☒	☐	A description of all covariates tested
☐	☒	A description of any assumptions or corrections, such as tests of normality and adjustment for multiple comparisons
☐	☒	A full description of the statistical parameters including central tendency (e.g. means) or other basic estimates (e.g. regression coefficient) AND variation (e.g. standard deviation) or associated estimates of uncertainty (e.g. confidence intervals)
☐	☒	For null hypothesis testing, the test statistic (e.g. *F*, *t*, *r*) with confidence intervals, effect sizes, degrees of freedom and *P* value noted<br>*Give P values as exact values whenever suitable.*
☒	☐	For Bayesian analysis, information on the choice of priors and Markov chain Monte Carlo settings
☒	☐	For hierarchical and complex designs, identification of the appropriate level for tests and full reporting of outcomes
☒	☐	Estimates of effect sizes (e.g. Cohen's *d*, Pearson's *r*), indicating how they were calculated

Our web collection on statistics for biologists contains articles on many of the points above.

Software and code

Policy information about availability of computer code

Data collection	No software was used
Data analysis	Particle image velocimetry is done in MATLAB 2016a using the open source software PIVLab 2.31. All image analysis was done by ImageJ (updated 2017). Graphs are plotted using MATLAB 2016a or OriginPro 2017.<br>The custom codes for data visualization and plotting of traction force microscopy on MATLAB are deposited on Github (https://github.com/leanhphuong201/extrusionnatcomm)

For manuscripts utilizing custom algorithms or software that are central to the research but not yet described in published literature, software must be made available to editors/reviewers. We strongly encourage code deposition in a community repository (e.g. GitHub). See the Nature Research guidelines for submitting code & software for further information.

Data

Policy information about availability of data

All manuscripts must include a data availability statement. This statement should provide the following information, where applicable:
- Accession codes, unique identifiers, or web links for publicly available datasets
- A list of figures that have associated raw data
- A description of any restrictions on data availability

The data supporting the findings of this study are available within the article and Supplementary Information or from the corresponding author upon reasonable request. A reporting summary for this article is available as a Supplementary Information file. Source data are provided with this paper.

Field-specific reporting

Please select the one below that is the best fit for your research. If you are not sure, read the appropriate sections before making your selection.

[x] Life sciences [ ] Behavioural & social sciences [ ] Ecological, evolutionary & environmental sciences

For a reference copy of the document with all sections, see nature.com/documents/nr-reporting-summary-flat.pdf

Life sciences study design

All studies must disclose on these points even when the disclosure is negative.

Sample size	While no statistical methods were used to predetermine sample size, we used the same range of data size (n > 15 extrusion events) as previous experiences and publications in the study of epithelia cell extrusion and wound healing using confocal live imaging (some examples: Kocgozlu, et al. (2016), Curr Biol; Ravasio et al., (2015), Nat Comm; Teo et al., (2020), Dev Cell ; Chen et al. (2019), Nat Phys.In all cases, the sample size used is more than necessary to get statistically significant results. Further information regarding exact data sample size can be found in the figure legends.
Data exclusions	We did not exclude any data from the analysis
Replication	Experiments were repeated in at least 2 different dates (denoted as replicate number m in the figures) with different samples point (denoted as number n in the figures). Replicates for each event pooled from different batches of experiments are represented by replicate number n. All attempts from replications are successful.
Randomization	Randomization is not relevant for our study. We treat each extrusion event as individual.
Blinding	Blinding is not relevant for our study as we did not include any animal works or clinical trials that involve the biased response of the group against the treatment. We used measurable quantity to determine phenotypes. Samples were processed and allocated according to specific experimental conditions and treatments (for e.g. specific knock-out and drug treatment).

Reporting for specific materials, systems and methods

We require information from authors about some types of materials, experimental systems and methods used in many studies. Here, indicate whether each material, system or method listed is relevant to your study. If you are not sure if a list item applies to your research, read the appropriate section before selecting a response.

Materials & experimental systems

n/a	Involved in the study	
[ ]	[x]	Antibodies
[ ]	[x]	Eukaryotic cell lines
[x]	[ ]	Palaeontology
[x]	[ ]	Animals and other organisms
[x]	[ ]	Human research participants
[x]	[ ]	Clinical data

Methods

n/a	Involved in the study	
[x]	[ ]	ChIP-seq
[x]	[ ]	Flow cytometry
[x]	[ ]	MRI-based neuroimaging

Antibodies

Antibodies used	Myosin IIA was stained by using Rabbit Anti-myosin IIA (Sigma M8064) 1:100. Paxillin was stained by using Rabbit Monoclonal anti-Paxillin [Y113] (Abcam ab32084) 1:100. Actin filaments were stained with Alexa Fluor® 568 Phalloidin (Cat# A12380, Life Technologies) 1:100 or Alexa Fluor® 488 Phalloidin (Cat# A12379, Life Technologies) 1:100 or Alexa Fluor® 647 Phalloidin (Cat# A22287, Life Technologies) 1:100. Secondary antibody is Goat Anti-rabbit IgG Alexa FluorR 568 (Cat# A-11011, Life Technologies) 1:100. Nucleus was labelled with Hoechst 33342 at 1µg/mL concentration. SiR Actin was used at 1:10000 concentration for live imaging (Cytoskeleton CY-SC001).
Validation	All antibodies were commercially obtained and used based on validation from the manufacturer. In particular:<br>Rabbit Anti-myosin IIA (Sigma M8064) were validated on MDCK (canine) and immunofluorescent staining.<br>Rabbit Monoclonal anti-Paxillin [Y113] (Abcam ab32084) were validated on mouse, rat, human (mammalian cells) and immunofluorescent staining.

Eukaryotic cell lines

Policy information about cell lines

Cell line source(s)	MDCK was obtained from ATCC
Authentication	None was authenticated
Mycoplasma contamination	Cell lines were tested for mycoplasma for every 3 months. Cells are clear of mycoplasma
Commonly misidentified lines (See ICLAC register)	none
