# Supplementary material for: Adhesion-mediated heterogeneous actin organization governs apoptotic cell extrusion
Source: Nat Commun. 2021 Jan 15;12:397. doi: 10.1038/s41467-020-20563-9 (PMC7810754; doi:10.1038/s41467-020-20563-9)
Supplement: Supplementary file 2 — Peer Review File [file 41467_2020_20563_MOESM2_ESM.pdf]

Reviewers' comments:

Reviewer #1 (Remarks to the Author):

This manuscript studies extrusion of cells from a confluent monolayer. The major point is that cells surrounding the extruding cell close the hole formed by the extruding cell using a combination of purse string- and lamellipodia-based force generation mechanisms, which contrasts with many studies focusing only on the purse string mechanism. Hindering both these mechanisms reduces the probability of successful extrusion and the rate of closing the area left by the extruded cell. Hindering one mechanism also reduces probability and rate, but to a lesser extent, suggesting that the two mechanisms are complementary. In general, the experimental design and data shown in the figures support the authors' conclusions, but there are some issues with the presentation of text and data and some technical issues with the experiments and interpretation. Specific comments follow, approximately in order of importance.

1. A theoretical model is provided at the back of the supplementary information. The description of the model in the main text is very brief, without enough information to fully understand the model. Additionally, it is unclear what the model adds—the model shows a matching between experiments and theory, but the model does not give new insights that are not already present in the experimental data. In fact, the authors' conclusions can be made based on the experimental data alone, without need for the model. It is recommended to remove the model.

2. There are some questions on traction force data in figure 4. The data show traction forces on cells cultured over nonadhesive patches. However, the images in d and e show nonzero traction force underneath the nonadhesive patches, especially for D = 15 um. If cells cannot adhere to the patches, shouldn't the traction forces be zero? Additionally the term "total traction force" is not defined. Does "total traction force" refer to the magnitude of the vector? The orientation data in 4f show a change in direction for D = 30 compared to 15 or 10 um, but the color plots in 4e do not show a change in color. The text on page 10 states "forces corresponded to lamellipodia protrusion generally pointed outwards from extrusion site, while those corresponded to partial actin cables are tangential to the cables (Supplementary Fig 11a)." The data in Supplementary Fig. 11a do not clearly support this claim. On page 11, the authors suggest that the extrusion requires a mean traction force of 200-400 Pa, but this suggestion does not explain the difference in extrusion probability on the patches of different size because Supplementary Figure 12 shows that the biggest patches (30 um) have average traction forces that are between the forces of the other patch sizes. Moreover, it is unclear whether traction force magnitude is a useful measure, because forces in the radial direction would appear to be more related to the extrusion. A substantial revision of the analysis and presentation of the traction data is needed.

3. For some supplemental figures, a paragraph of text is used to describe them. If a full paragraph is required, the figures are important and should be moved to the main text. If the figures are not important enough to be in the main text, then the amount of text used to describe them should be reduced.

4. Figure 2 shows that the actomyosin rings associated with the purse string mechanism are typically oriented at an angle. That figure also shows inhomogeneity in fluorescent labeling of the purse strings. Did the authors rule out that the inhomogeneity is caused, at least in part, but the fact that the purse string is at an angle?

5. One the bottom of page 5, it states "the inhomogeneity in actomyosin II staining cable decreased with the intensity of caspase-3 (Fig. 2b, Supplementary Fig. 4b)". In contrast, neither figure shows a clear inverse correlation between the two.

6. In figure 2, authors measure inhomogeneity using two metrics, mean / 90th percentile and standard deviation / mean. It seems odd to use one definition wherein mean is the numerator and

another wherein mean is the denominator. The authors should pick one and use it consistently.

7. The abstract states "The early presence of basal lamellipodia protrusion was crucial not only in basal sealing of the extrusion site but also in orienting the actomyosin purse-string…" The word crucial seems like an overstatement, given that in the experiments with blebbistatin and SMIFH2, extrusion still occurred about half the time.

8. There are typos in the text, including grammatical issues and references to figure panels that do not exist. Additional proofreading is recommended.

Reviewer #2 (Remarks to the Author):

The paper by Le et al describes a new dual mechanism for regulating the extrusion of apoptotic cells from epithelial monolayers. While previous studies have focused on the role of a uniform multicellular purse-string that surrounds the apoptotic cell and promotes extrusion, the authors find a heterogeneous actin network in the neighbors surrounding the apoptotic cell. They describe how two distinct cellular processes, lamellipodia protrusions and contractile actomyosin cables, are coordinated to regulate apoptotic cell extrusion. The authors dissect the temporal progression through extrusion by modulating the strength of the adherens junctions at cell-cell contacts using alpha-catenin mutants and also changing the patterns of substrate for cell-matrix adhesion. The authors uncouple these cellular processes, and show a sequential progression of lamellipodia protrusions and then actomyosin ring contraction to control successful extrusion. The force patterns generated during extrusion were deduced from the experimental models and combined with theoretical modeling to delineate the contribution of the respective forces exerted by the purse-string versus that of lamellipodia protrusions. Together, the authors conclude that adhesion mediated heterogenous actin organization governs the process of apoptotic cell extrusion in epithelia. This is an interesting paper and the findings further our understanding of how the process of cell extrusion is regulated to eject dying cells while maintaining epithelial integrity. Overall, I find that the authors' conclusions are supported by the data and the experiments are thoroughly controlled. Therefore, it would be of interest to the readership of the Nature Communications.

I do, however, have a couple of small points that should be addressed prior to publication.

The authors' provide data that basal lamellipodia protrusions "emerged in response to apoptosis and caspase 3 activation". Do the basal lamellipodia protrusions form when caspase 3 or apoptosis is inhibited?

It would also be useful to know if the authors also observed actomyosin rings in the apoptotic extruding cells themselves, and if so, were they heterogeneous or homogenous?

Minor points:

Arrowheads on Fig 3 labeling protrusion would be helpful.

Image in Fig 3a is labeled 30min, reference in text on pg 7, line 197 is for 33min.

Careful proofreading of the manuscript is necessary – Eg. "Laser ablation performed these regions in acatKD cells induced junctional recoil much 2 time slower than the one observed in WT cells" pg 5 line 200-202

The acronym "CCJ" should be defined the first time it is used in the text pg 8 line 227

Reviewer #3 (Remarks to the Author):

Phuong Le &al perform an analysis of cellular structures and forces involved in apoptotic cell extrusion, in MDCK monolayers. The authors focus on the relative role of an apical junctional cable around the extruded cell and basal lamellipodia. They find that the extruded cell constricts more quickly basally than apically, whereas the apical and basal constriction rates are similar when lamellipodia are inhibited. The apical actin cable surrounding the extruding cell is not continuous, but seems to drive individual cell junction constriction. The authors then argue that a temporal sequence exists for individual cells where lamellipodial formation is followed by apical myosin recruitment. They find that inhibiting alpha-actinin leads to increased lamellipodia formation at the expense of constriction by an apical cable. The authors then explore constriction in the absence of lamellipodia by using patterned adhesive substrate. They find radial actin cables connecting the boundary of the extruding cell to adhesion patches. For large enough patterns of inhibited adhesion, cell extrusion tends to fail. The authors then perform traction force microscopy and compare the patterns of forces for different pattern sizes. A model of cell extrusion, predicting the evolution of the extruded cell radius taking into account several force contribution, is then discussed. The authors then report patterns of traction force microscopy for extrusions on patterns with contours of varying curvature.

In general the manuscript presents a remarkably large number of results and made a serious effort to quantify the process of cell extrusion. However I thought it was difficult to extract a coherent picture from it.

Related to this, I thought that the model was interesting but could have been used to support a more clear dissection of the mechanisms behind extrusion. At the moment a set of reasonable, but arbitrarily parameters is used to produce a graph in Fig. 5c, showing that the probability of closure increases with the apical line tension and is lower for larger cells. While I can see that there is value in exploring the generic behaviour of the model, here the conclusions are quite straightforward (two constricting forces are more efficient than one), and it is not clear what quantitative insight is brought (it seems that there is no fitting to data that would return one of the parameters, for instance). I think the manuscript would be improved if the model was used to help bringing a coherent picture of how closure occurs. For instance, it is possible to predict or discuss the relative apical and basal constriction rates; and more generally the relationship between apically and basally generated forces? What is the role of the radial cables in the 3D force balance? Can the data on traction force microscopy and recoil velocity after laser ablation be used to constrain the model? Or can the experiments with patterns of adhesion with positive and negative curvature be used to make comparisons between model predictions and observations?

Some of the experimental results are interesting but it is unclear what information they bring. What is the interpretation, for instance, of the varying traction force microscopy pattern observed as a response to lamellipodia formation and actin cable formation? It is also remarkable that the curvature of the adhesion pattern influences the type of extrusion force; but how can this observation be explained?

Other comments:
- I did not understand how the authors interpret the tilting of actomyosin cables?
- Maybe this is a typo, but I did not understand why the viscosity eta introduced in Eq. 2 is absent in the pressure equation in the text before Eq. 4. If eta is a bulk viscosity, it should contribute a term \partial_{\gamma} v_{\gamma} \delta_{\alpha\beta} to the stress tensor in Eq. 2? And if it is a shear viscosity as in Eq.2, it should appear in the expression for the pressure?
- I am also not sure about the status of the radial fibres; it looks like they are anchored to the substrate? Then the entire epithelium can not flow relative to the substrate, as described in the model? More generally the 3D distribution of these cables was not clear to me.

- In Fig. S11a, how are forces correlated to actin cables and to lamellipodia distinguished in practice?
- in page 12 in the main text: "Analytically, we find that the cell extrusion fails whenever the size of the non-adhesive patch is bigger than a critical radius "; I was not sure which section is referred to?
- The authors report values of traction stresses acting on the substrate as quantifying forces exerted by the lamellipodium and the actin cable; however because the tissue itself is also supporting stresses, it seems to me this is an oversimplification; i.e there is a limit where all the stress would be supported by the tissue and there would not be any traction force?
- In general I thought that the manuscript was often referring to important pieces of data in the supplementary. I would suggest to transfer some this data to the main text.

Reviewers' comments:

Reviewer #1 (Remarks to the Author):

This manuscript studies extrusion of cells from a confluent monolayer. The major point is that cells surrounding the extruding cell close the hole formed by the extruding cell using a combination of purse string- and lamellipodia-based force generation mechanisms, which contrasts with many studies focusing only on the purse string mechanism. Hindering both these mechanisms reduces the probability of successful extrusion and the rate of closing the area left by the extruded cell. Hindering one mechanism also reduces probability and rate, but to a lesser extent, suggesting that the two mechanisms are complementary. In general, the experimental design and data shown in the figures support the authors' conclusions, but there are some issues with the presentation of text and data and some technical issues with the experiments and interpretation. Specific comments follow, approximately in order of importance.

1. A theoretical model is provided at the back of the supplementary information. The description of the model in the main text is very brief, without enough information to fully understand the model.

We agree with the referee that the link between theory and experiments was insufficiently put forward in the main text of the paper. We think we now have corrected this point in the new version of the manuscript by incorporating a detailed description of the model in the manuscript (pg13-4, line 335-387). Please see below answer for more details.

Additionally, it is unclear what the model adds—the model shows a matching between experiments and theory, but the model does not give new insights that are not already present in the experimental data. In fact, the authors' conclusions can be made based on the experimental data alone, without need for the model. It is recommended to remove the model.

We would like to clarify the interest of the theory in this context. Several competing mechanisms could lead to cell extrusion. For instance, the fact that cell extrusion could fail on non-adherent substrate was indicative that cell extrusion was related to forces generated by the surrounding tissue. Therefore, we clarified the model in the revised version of the manuscript (See revised main text and for more details, Supplementary material). In summary, based on active fluid theory, the model thus provides a phase diagram of cell extrusion processes that can be compared to experiments (Figure 8) and helped to determine the relative strengths of the different processes during cell extrusion. Our theoretical approach leads a unified framework explaining why cell extrusion necessarily succeed on adherent substrates, but not on non-adherent and also relates the strength of the acto-myosin cable to cell-cell junction strength. The latter has been further confirmed by the impact of α-catenin KD on actin tension, revealed by ablation experiments (Figure 3 c-f).

Furthermore, we notice that the time required for the extrusion process is highly variable. A similar variability is observed in the time required to achieve wound closure in epithelial

tissues; this suggested us that some similarity with the mechanisms at stake in wound closure. We therefore considered the theoretical models developed in following two references (Cochet-Escartin, Ranft, Silberzan, & Marcq, 2014) and (V. Nier et al., 2015). We further clarify the similarity and differences between the wound closure and cell extrusion dynamics.

As in wound-healing experiments, our adapted stochastic model is suggestive of a counter-intuitive result: cell extrusion does occur on non-adherent substrates, potentially even in regimes where, on average, mechanical forces would prevent it to happen; indeed, our model, suggesting that extrusion could occur through the accumulation of favorable mechanical fluctuations (ξ term), predicts large variability in the extrusion time. In other words, the presence of mechanical fluctuations may lead to the completion of the extrusion process, even in a phase space of parameters where deterministic forces would prevent it from occurring; it would therefore be of interest to check whether α-catenin may not just reduce the overall contractility, but also the level of contractility fluctuations.

While failure to complete wound closure on non-adherent substrate was interpreted in terms of an elastic tissue response in Cochet-Escartin 2014, our model suggests that failure to complete cell extrusion could be due the existence of apico-basal cables under tension. Our experiments showed that, indeed, apico-basal cables are indeed under large tension, with values compatible with model estimates.

Though inspired by previous models for wound closure, our model is more general than the previous models developed in (Cochet-Escartin et al., 2014) and (V. Nier et al., 2015). The wound-closure theoretical framework needs to be significantly modified since the length scales involved in cell extrusion are significantly lower than during the wound closure processes while time scales are comparable, meaning that the origin and strength of the forces involved in the closing dynamic are different.

2. There are some questions on traction force data in figure 4. The data show traction forces on cells cultured over non-adhesive patches. However, the images in d and e show non-zero traction force underneath the non-adhesive patches, especially for D = 15 um. If cells cannot adhere to the patches, shouldn't the traction forces be zero?

Several factors could contribute to the fact that there were traction forces detected in the non-adhesive region:
- The traction force is carried out on continuous elastic substrate on which the propagation of deformations is relatively long range and based on Fourier transform of the displacement of fiducial markers on the substrate. Strong pulling on the edges of non-adhesive patches may result in bead displacements even in the middle of the small non-adhesive patches, conducting therefore to non-zero forces inference (See for instance, Schwarz et al. BBA Mol Cell Research, 2015, Fig 2). It is however technically challenging to replace the force inference method.
- Alternatively, small bead displacement on the non-adhesive region may result from the non-specific sliding of the dying cell on the substrate could result in small forces detection.

Additionally the term "total traction force" is not defined. Does "total traction force" refer to the magnitude of the vector?

We clarified this point in the revised version of the manuscript (page 10, line 247) and in Fig.5d (previously Fig.4d) caption.

The orientation data in 4f show a change in direction for D = 30 compared to 15 or 10 um, but the color plots in 4e do not show a change in color. The text on page 10 states "forces corresponded to lamellipodia protrusion generally pointed outwards from extrusion site, while those corresponded to partial actin cables are tangential to the cables (Supplementary Fig 11a)."

We thank the reviewer for her/his remark. We made a mistake on labeling the figure. The label on Fig4f (In the current version it is Fig.5f) should be reversed. Fig.5f: From left to right: D = 30 µm, 15 µm, and 10 µm. Force orientation on 10 µm is more anisotropic than forces on D = 15 µm and D = 30 µm, which are generally pointing inwards.

The data in Supplementary Fig. 11a do not clearly support this claim. On page 11, the authors suggest that the extrusion requires a mean traction force of 200-400 Pa, but this suggestion does not explain the difference in extrusion probability on the patches of different size because Supplementary Figure 12 shows that the biggest patches (30 um) have average traction forces that are between the forces of the other patch sizes. Moreover, it is unclear whether traction force magnitude is a useful measure, because forces in the radial direction would appear to be more related to the extrusion. A substantial revision of the analysis and presentation of the traction data is needed.

We thank the reviewer to the comments. To make the message clearer, we presented the data in terms of Tension per unit length – derived from Radial Traction Force and Work done by Radial Traction Force component (Supplementary Figure 11).
For extrusion on the non-adhesive patch, we used force balance principle to calculate the force exerted by purse-string. For the total work done to move the cell edge across the non-adhesive area of radius R, without lamellipodia protrusion, the force F_{radial} needs to be large enough to overcome the resistivity of the bulk:
$dW_{radial} = F_{radial}.dr \geq \gamma. (2\pi R)/dr$ (Only radial component contributes to closure).
At non-adhesive patch size of D = 30 µm, ,since extrusion always fails, we could estimate the force exerted by purse-string to drive extrusion by identifying the range at which the Work done changed sign and the area expanded (range between the dashed line on Supplementary Figure 11a, third panel). As such, the tension induced by purse-string can be estimated to be $\gamma = 6.5 \pm 1.4$ nN/ µm (Supplementary Fig. 11a, second panel).
Such estimation is validated again on extrusion on non-adhesive patch size of D = 15 µm (Supplementary Fig. 11b)**.** Note that between this range of forces, the purse-string produces non-zero Work and as such, extruding area is reduced linearly before reaching flat ends.
On patch size of D = 10 µm, we observed that the forces produced by purse-string only started increasing and the corresponding Work started to be non-zero only when the extruding area falls below the patch size (from 40 min onwards, r = 2-5 µm). This suggested that contractile purse-string did not contribute to extrusion until the later phase (Supplementary Fig. 11c).

3. For some supplemental figures, a paragraph of text is used to describe them. If a full paragraph is required, the figures are important and should be moved to the main text. If the figures are not important enough to be in the main text, then the amount of text used to describe them should be reduced.

We moved some supplementary figures in the old version to the main figures and also modified the order of the main figures. Please refer to the detailed table below for the figure order changes.

Original manuscript	Revised manuscript
Supplementary Fig.5b'	Fig.2f
Supplementary Fig.8a-c	Fig.3c-f
Supplementary Fig.8d-d'	Fig.4a-b
Fig.3c-d'	Fig.4c-e
Supplementary Fig.8e	Fig.4f
Fig.4	Fig.5
Supplementary Fig.9	Supplementary Fig.8
Supplementary Fig.10	Supplementary Fig.9
Supplementary Fig.11	Supplementary Fig.10
Supplementary Fig.13a-c	Fig.6 b-d
Fig.5b	Fig.6e
Fig.6	Fig.7
Fig.5c	Fig.8e-f

4. Figure 2 shows that the actomyosin rings associated with the purse string mechanism are typically oriented at an angle. That figure also shows inhomogeneity in fluorescent labeling of the purse strings. Did the authors rule out that the inhomogeneity is caused, at least in part, but the fact that the purse string is at an angle?

To rule out that the heterogenous purse-string observed at the early event was a full purse-string that was tilted, we averaged the actin or myosin intensity from z = 1 µm to z = +6 µm from the basal plane. The representations of these images are also shown in the average projection (Fig. 2a).

5. One the bottom of page 5, it states "the inhomogeneity in actomyosin II staining cable decreased with the intensity of caspase-3 (Fig. 2b, Supplementary Fig. 4b)". In contrast, neither figure shows a clear inverse correlation between the two.

We thank the reviewer for this point. We re-analyzed the calculation of this set of experiments. We realized that previously we did not normalize for the myosin background for this set of experiments. We corrected for this by first subtracting the myosin background intensity then afterwards, taking inhomogeneity mean/90th percentile. Our revised data show that the inhomogeneity of the cable indeed decreases with caspase intensity (Fig.2b).

6. In figure 2, authors measure inhomogeneity using two metrics, mean / 90th percentile and

standard deviation / mean. It seems odd to use one definition wherein mean is the numerator and another wherein mean is the denominator. The authors should pick one and use it consistently.

We thank the reviewer for this remark. We removed the standard deviation/mean in Fig.2 and used only mean/ 90th percentile to measure inhomogeneity in the revised version of the manuscript.

7. The abstract states "The early presence of basal lamellipodia protrusion was crucial not only in basal sealing of the extrusion site but also in orienting the actomyosin purse-string…" The word crucial seems like an overstatement, given that in the experiments with blebbistatin and SMIFH2, extrusion still occurred about half the time.

We rephrased the sentence in the abstract (page 2) into "The early presence of basal lamellipodia protrusion participated both in basal sealing of the extrusion site and in orienting the actomyosin purse-string at the later stage of extrusion."

8. There are typos in the text, including grammatical issues and references to figure panels that do not exist. Additional proofreading is recommended.

We proofread the whole manuscript again.

Reviewer #2 (Remarks to the Author):

The paper by Le et al describes a new dual mechanism for regulating the extrusion of apoptotic cells from epithelial monolayers. While previous studies have focused on the role of a uniform multicellular purse-string that surrounds the apoptotic cell and promotes extrusion, the authors find a heterogeneous actin network in the neighbors surrounding the apoptotic cell. They describe how two distinct cellular processes, lamellipodia protrusions and contractile actomyosin cables, are coordinated to regulate apoptotic cell extrusion. The authors dissect the temporal progression through extrusion by modulating the strength of the adherens junctions at cell-cell contacts using alpha-catenin mutants and also changing the patterns of substrate for cell-matrix adhesion. The authors uncouple these cellular processes, and show a sequential progression of lamellipodia protrusions and then actomyosin ring contraction to control successful extrusion. The force patterns generated during extrusion were deduced from the experimental models and combined with theoretical modeling to delineate the contribution of the respective forces exerted by the purse-string versus that of lamellipodia protrusions. Together, the authors conclude that adhesion mediated heterogenous actin organization governs the process of apoptotic cell extrusion in epithelia. This is an interesting paper and the findings further our understanding of how the process of cell extrusion is regulated to eject dying cells while maintaining epithelial integrity. Overall, I find that the authors' conclusions are supported by the data and the experiments are thoroughly controlled. Therefore, it would be of interest to the readership of the Nature Communications.

We thank the reviewer for his/her positive comments on our manuscript.

I do, however, have a couple of small points that should be addressed prior to publication. The authors' provide data that basal lamellipodia protrusions "emerged in response to apoptosis and caspase 3 activation". Do the basal lamellipodia protrusions form when caspase 3 or apoptosis is inhibited?

Epithelial cells exhibit random cryptic lamellipodia even without extrusion (see for instance our recent paper (Jain et al., 2020)). When we looked at PBD signals in the monolayer as an indicator of lamellipodia protrusion (Supplementary Fig. 1e-e'), we observed that even without cell death (indicated by caspase activation), there were still random lamellipodia indicated by spontaneous flaring of PBD signals (Supplementary Fig. 1e, arrowheads). So we do not expect to inhibit lamellipodia protrusions by inhibiting apoptosis. However, random lamellipodia protrusions in the bulk of the monolayers are instantaneous and do not persist: they last for 3-6 minutes which are much shorter than lamellipodia protrusion in response to apoptosis (51 min, Supplementary Fig. 1a). In addition, lamellipodia protrusions in the case of extrusion are directed toward the site of extrusion (Supplementary Fig.1a-b).

It would also be useful to know if the authors also observed actomyosin rings in the apoptotic extruding cells themselves, and if so, were they heterogeneous or homogenous?

Previous studies cited in our paper (Gagliardi et al., 2018; Kuipers et al., 2014) describe the formation of an actomyosin ring inside the dying cells. However, the presence of this ring was not the focus of this paper, so we did not design experiments to characterize this ring.

Moreover, it could be technically challenging to clearly distinguish and characterize actomyosin rings formed by extruding and neighboring cells. Our study is more focused on the role of neighbors. We agree that it would be interesting in the future to study the actin organization and dynamics within the dying cell, but we think that this is currently beyond the scope of this manuscript.

Minor points:
Arrowheads on Fig 3 labeling protrusion would be helpful. Image in Fig 3a is labeled 30min, reference in text on pg 7, line 197 is for 33min.
Careful proofreading of the manuscript is necessary – Eg. "Laser ablation performed these regions in acatKD cells induced junctional recoil much 2 time slower than the one observed in WT cells" pg 5 line 200-202.
The acronym "CCJ" should be defined the first time it is used in the text pg 8 line 227.

We thank the reviewer for the comments. We have fixed these points. In particular:
- We added the arrowheads to label the protrusion Fig.3a. The reference to the figure was changed to 30 min in pg8, line 163.
- We defined the acronym "CCJ" for the first time on pg8, line 169.

Reviewer #3 (Remarks to the Author):

Phuong Le &al perform an analysis of cellular structures and forces involved in apoptotic cell extrusion, in MDCK monolayers. The authors focus on the relative role of an apical junctional cable around the extruded cell and basal lamellipodia. They find that the extruded cell constricts more quickly basally than apically, whereas the apical and basal constriction rates are similar when lamellipodia are inhibited. The apical actin cable surrounding the extruding cell is not continuous, but seems to drive individual cell junction constriction. The authors then argue that a temporal sequence exists for individual cells where lamellipodial formation is followed by apical myosin recruitment. They find that inhibiting alpha-actinin leads to increased lamellipodia formation at the expense of constriction by an apical cable. The authors then explore constriction in the absence of lamellipodia by using patterned adhesive substrate. They find radial actin cables connecting the boundary of the extruding cell to adhesion patches. For large enough patterns of inhibited adhesion, cell extrusion tends to fail. The authors then perform traction force microscopy and compare the patterns of forces for different pattern sizes. A model of cell extrusion, predicting the evolution of the extruded cell radius taking into account several force contribution, is then discussed. The authors then report patterns of traction force microscopy for extrusions on patterns with contours of varying curvature.

In general the manuscript presents a remarkably large number of results and made a serious effort to quantify the process of cell extrusion. However I thought it was difficult to extract a coherent picture from it.

Related to this, I thought that the model was interesting but could have been used to support a more clear dissection of the mechanisms behind extrusion. At the moment a set of reasonable, but arbitrarily parameters is used to produce a graph in Fig. 5c, showing that the probability of closure increases with the apical line tension and is lower for larger cells. While I can see that there is value in exploring the generic behaviour of the model, here the conclusions are quite straightforward (two constricting forces are more efficient than one), and it is not clear what quantitative insight is brought (it seems that there is no fitting to data that would return one of the parameters, for instance). I think the manuscript would be improved if the model was used to help bringing a coherent picture of how closure occurs. For instance, it is possible to predict or discuss the relative apical and basal constriction rates; and more generally the relationship between apically and basally generated forces? What is the role of the radial cables in the 3D force balance? Can the data on traction force microscopy and recoil velocity after laser ablation be used to constrain the model? Or can the experiments with patterns of adhesion with positive and negative curvature be used to make comparisons between model predictions and observations?

We thank the reviewers for his/her positive comments on our study and suggestions to improve our manuscript. In the revised version, we improved the data representation and the writing to clarify our message and the insights brought from the model and experiments.

First, we presented traction force data as the estimate for the tension exerted by purse-string and its changes as the function of time reflect extrusion closure. We determined the timescale and magnitude force range at which extrusion is successful (Fig.8a-d and Supplementary Fig. 11). The value is comparable with the same one reported in the model. The purse-string tension is experimentally estimated to be 6.5 ± 1.4 nN/µm for WT (corresponding to 6-8 nN/µm in the model) and less than 1 nN/µm for αcatKD (corresponding to 0 nN/µm purse-string contractility in the model).

Secondly, we showed experimentally the presence of an apical-basal actin cables, which is under tension (Supplementary Fig.8b-c). This helps us to establish the term σ_{μ} to account for the stress exerted through apico-basal actin cables. This term helps us to establish the interconnectivity between apical and basal side.
From the model, we showed that the presence of fluctuations may lead to the completion of the extrusion process, even in a phase space of parameters where deterministic forces would prevent it from occurring. This is non-intuitive and has not been shown in previous theoretical framework on wound healing, which also addresses lamellipodia and purse-string but on longer length scale, yet similar timescale. The model also suggested that α-catenin may not just reduce the overall contractility, but also the level of contractility fluctuations, which could not be established without the analytical model. We used experiments with different α-catenin mutants seeded on different non-adhesive patch sizes to compare with the numerical simulation derived from the model. The trend obtained from numerical solutions varying the strength of the purse-string contractility closely follows the trend observed in experiments with different αcat mutant background for cells on non-adhesive patches (see Fig.8e-f).
Lastly, on Fig.7, we showed that using asymmetric non-adhesive patches, we could have conditions in which lamellipodia and purse-string were both present but spatially separated. Under this condition, we were able to measure the traction stresses exerted by each structure to further support the model. Also, one main assumption of the model is that the protrusive stress by lamellipodia is a constant, and our traction force analysis clearly supported this (Fig.7d).

Some of the experimental results are interesting but it is unclear what information they bring. What is the interpretation, for instance, of the varying traction force microscopy pattern observed as a response to lamellipodia formation and actin cable formation? It is also remarkable that the curvature of the adhesion pattern influences the type of extrusion force; but how can this observation be explained?

We corrected the manuscript, especially Supplementary Fig.10 regarding the variation of traction force pattern as a response to lamellipodia formation and actin cable formation. The force measurement on fully adhesive substrate show that forces increase at extrusion sites and during the process when compared to control areas. We observe both inward and outward-pointing forces that suggest a dual mechanism driven by cell protrusion and purse string as previously shown in our studies on wound healing and epithelial gap closure (Brugués et al., 2014; Chen et al., 2019) (Supplementary Fig. 10).
As pointed out by the reviewer, we used non-adhesive patterns with either isotropic (Fig.5) or anisotropic shape (Fig.7) and measured the corresponding forces to each mechanism to determine the contribution of each mechanism. As previously reported in model wound assays (Ravasio et al., 2015) , we showed that the curvature of adhesive patterns could favor lamellipodia protrusion or purse string mechanism. Preferential formation of a cable at the negative-curvature is observed whereas lamellipodia protrusion was dominant at the positive curvature side leading to different force patterns. We have now clarified this in the revised version of the manuscript (p. 12-13, line 310 - 332).

Other comments:
- I did not understand how the authors interpret the tilting of actomyosin cables?

We thank the referee for pointing out this point, since tilting of the actomyosin cables is a crucial element of the theoretical framework. Tilting is caused by the relative position at the extruding/neighboring cells of focal points at the basal level and E-cadherin adhesions at the apical level.
From the starting point of extrusion till basal closure, the non-uniform formation of lamellipodia protrusion leads to asymmetric losing of apical-basal polarity. This together with non-uniform distribution of contractile actomyosin purse-string results in the tilted full purse-string at the later time point, when basal closure is complete. We postulated that the tilting of the full ring at the end stage of extrusion is due to two factors: non-uniform formation of contractile actin cable at apical stage and lamellipodia protrusion that delays myosin enrichment to form contractile cables.
The representative example on Fig.1a illustrates this point. Cells that formed lamellipodia lost their apical-basal polarity (Fig.1a, cell #1, top view and side view), which resulted in the pulling of its neighbor's intercellular junction to basal plane (cell #2,3 part of cell #4). However, the cell #4 retained its apicobasal polarity until later time point when basal closure is almost complete (18 min), cell #4 started to form actomyosin cable. As a result, the final full purse-string has parts connected to the basal. We further illustrated the distribution of F-actin at protrusive edges versus contractile myosin at partial purse-string by the example on Supplementary 6.

- Maybe this is a typo, but I did not understand why the viscosity eta introduced in Eq. 2 is absent in the pressure equation in the text before Eq. 4. If eta is a bulk viscosity, it should contribute a term \partial_{\gamma} v_{\gamma} \delta_{\alpha\beta} to the stress tensor in Eq. 2? And if it is a shear viscosity as in Eq.2, it should appear in the expression for the pressure?
Indeed, this technical point was insufficiently explained in the previous version of the Supplementary Material. We have added further explanation in the current version (Theoretical model, supplementary text line 29-33). The expression of the stress, Eq. (2), includes a viscous dissipation $\eta v_{\alpha\beta}$, where

$$v_{\alpha\beta} = \frac{1}{2}(\partial_\alpha v_\beta + \partial_\beta v_\alpha)$$

is a symmetric shear rate; indeed, we ought to have mentioned the important equality between the symmetric shear rate and the deviatoric shear rate $\tilde{v}_{\alpha\beta} = v_{\alpha\beta}$ that is due to the incompressibility assumption; up to that point, we have closely followed the arguments and notation of Cochet-Escartin Biophys. J. 2014.

- I am also not sure about the status of the radial fibres; it looks like they are anchored to the substrate? Then the entire epithelium cannot flow relative to the substrate, as described in the model? More generally the 3D distribution of these cables was not clear to me.

We observe that radial fibers are connected to focal adhesions; yet attachment over short time scale (which is less than minute - (Salbreux, Charras, & Paluch, 2012)) does not prevent flows of fibers over longer time scale > 10 min (Salbreux et al., 2012) through constant turnover of the molecules that are constitutive of the focal adhesions complexes. On non-adhesive substrates, we observe large radial fibers that span from the basal side to the apical side; in order to make this point clearer, we added a sketch in Figure 8a-d. These large-scale

structures only occur at the extruding area, whose scale is small compared to the whole epithelium. These radial fibers are interconnected between the substrate and the purse-string, which is visualized in Fig. 5b, Supplementary Fig.8d-d' and the illustration on Fig.8d.

- In Fig. S11a, how are forces correlated to actin cables and to lamellipodia distinguished in practice?

In fact, it is hard to distinguish the forces corresponding to lamellipodia and purse-string solely based on traction forces on continuous substrate. The reason is first, there both mechanisms are present at such short length scale event. Second, there are fluctuation components as shown in the model which could contribute to these forces. Therefore, we used the non-adhesive patches (both isotropic shape and asymmetric shape with two kinds of curvature) as a way to discriminate lamellipodia and purse-string forces and infer the force parameters from there.

- in page 12 in the main text: "Analytically, we find that the cell extrusion fails whenever the size of the non-adhesive patch is bigger than a critical radius "; I was not sure which section is referred to?

It refers to the model explained in the Supplementary Information. This is clarified in the revised version of the manuscript in the sentence "We show that the cell extrusion fails whenever the size of the non-adhesive patch is bigger than a critical radius, which can be estimated by numerical simulation (pg 14, line 375-376, see Theoretical Appendix- Fig.SI Theory Fig.2)."

- The authors report values of traction stresses acting on the substrate as quantifying forces exerted by the lamellipodium and the actin cable; however because the tissue itself is also supporting stresses, it seems to me this is an oversimplification; i.e there is a limit where all the stress would be supported by the tissue and there would not be any traction force?

Here we directly measured the traction forces at the cell extrusion site. These forces are thus exerted at cell-substrate interactions. The stress in a cell monolayer is balanced by the traction forces the monolayer exerts on its substrate everywhere in space and time.
However, as rightly mentioned referee, traction forces could happen to be extremely low (e.g. tissues over a lipid bilayer). This is, however, not the case in the experiments we consider, whereby traction forces are sufficiently large to lead to significant bead deflections. Whenever tractions forces are large enough, it is possible to infer the supporting stress within the monolayer through the force balance relation

$$\nabla \sigma = -T$$

In particular, BISM (Vincent Nier et al., 2016) is based on such relation.

- In general I thought that the manuscript was often referring to important pieces of data in the supplementary. I would suggest to transfer some this data to the main text.

We have incorporated previous supplementary figures in the main text. The particular rearrangement can be found in the below table:

Original manuscript	Revised manuscript
Supplementary Fig.5b'	Fig.2f

Supplementary Fig.8a-c	Fig.3c-f
Supplementary Fig.8d-d’	Fig.4a-b
Fig.3c-d’	Fig.4c-e
Supplementary Fig.8e	Fig.4f
Fig.4	Fig.5
Supplementary Fig.9	Supplementary Fig.8
Supplementary Fig.10	Supplementary Fig.9
Supplementary Fig.11	Supplementary Fig.10
Supplementary Fig.13a-c	Fig.6 b-d
Fig.5b	Fig.6e
Fig.6	Fig.7
Fig.5c	Fig.8e-f

Brugués, A., Anon, E., Conte, V., Veldhuis, J. H., Gupta, M., Colombelli, J., . . . Trepat, X. (2014). Forces driving epithelial wound healing. *Nature Physics, 10*, 683. doi:10.1038/nphys3040

Chen, T., Callan-Jones, A., Fedorov, E., Ravasio, A., Brugués, A., Ong, H. T., . . . Ladoux, B. (2019). Large-scale curvature sensing by directional actin flow drives cellular migration mode switching. *Nature Physics, 15*(4), 393-402. doi:10.1038/s41567-018-0383-6

Cochet-Escartin, O., Ranft, J., Silberzan, P., & Marcq, P. (2014). Border forces and friction control epithelial closure dynamics. *Biophys J, 106*(1), 65-73. doi:10.1016/j.bpj.2013.11.015

Gagliardi, P. A., Somale, D., Puliafito, A., Chiaverina, G., di Blasio, L., Oneto, M., . . . Primo, L. (2018). MRCKalpha is activated by caspase cleavage to assemble an apical actin ring for epithelial cell extrusion. *J Cell Biol, 217*(1), 231-249. doi:10.1083/jcb.201703044

Jain, S., Cachoux, V. M. L., Narayana, G. H. N. S., de Beco, S., D’Alessandro, J., Cellerin, V., . . . Ladoux, B. (2020). The role of single-cell mechanical behaviour and polarity in driving collective cell migration. *Nature Physics, 16*(7), 802-809. doi:10.1038/s41567-020-0875-z

Kuipers, D., Mehonic, A., Kajita, M., Peter, L., Fujita, Y., Duke, T., . . . Gale, J. E. (2014). Epithelial repair is a two-stage process driven first by dying cells and then by their neighbours. *J Cell Sci, 127*(Pt 6), 1229-1241. doi:10.1242/jcs.138289

Nier, V., Deforet, M., Duclos, G., Yevick, H. G., Cochet-Escartin, O., Marcq, P., & Silberzan, P. (2015). Tissue fusion over nonadhering surfaces. *Proc Natl Acad Sci U S A, 112*(31), 9546-9551. doi:10.1073/pnas.1501278112

Nier, V., Jain, S., Lim, Chwee T., Ishihara, S., Ladoux, B., & Marcq, P. (2016). Inference of Internal Stress in a Cell Monolayer. *Biophysical Journal, 110*(7), 1625-1635. doi:https://doi.org/10.1016/j.bpj.2016.03.002

Ravasio, A., Cheddadi, I., Chen, T., Pereira, T., Ong, H. T., Bertocchi, C., . . . Ladoux, B. (2015). Gap geometry dictates epithelial closure efficiency. *Nat Commun, 6*, 7683. doi:10.1038/ncomms8683

Salbreux, G., Charras, G., & Paluch, E. (2012). Actin cortex mechanics and cellular morphogenesis. *Trends Cell Biol, 22*(10), 536-545. doi:10.1016/j.tcb.2012.07.001

REVIEWER COMMENTS

Reviewer #1 (Remarks to the Author):

After reading the revised manuscript and the rebuttal letter, the following questions remain:

Regarding the model:
- The main text describing the model has been revised, but there are still insufficient details in the main text. For example, the reader has to look to the supplemental information to see that the model assumes the cell layer is a viscous incompressible fluid. This assumption is important, because it is required to relate the stresses with the change in radius.
- My questions about the importance of the model have been partly addressed in the rebuttal letter, including comments responding to a similar question by reviewer 3. In short, the model does two things. 1. It shows how purse string contraction and nonadhesive patch size jointly affect extrusion, with findings being as expected and therefore demonstrating that the model can capture some experimental observations. 2. It shows that fluctuations in contraction can increase the probability of extrusion, which is a new result that builds on experimental findings. In the current version of the manuscript, the results on stress fluctuations are located in supplemental information, and the text describes the fluctuations at the end of the section on the model rather than at the start. The importance of this result would be clearer if the manuscript were reorganized to draw more attention to the idea of fluctuations.

Regarding the analysis of traction force:
- Nonzero traction forces are observed inside the nonadhesive patch. The rebuttal letter suggests that bead displacements inside the adhesive patch could cause nonzero tractions inside the patch. This is not necessarily true, as nonzero bead displacements in the center of the patch could exist even if no tractions are located inside the patch. However, limited spatial resolution could potentially cause tractions to exist in a region slightly larger than the boundary of the cells. However, if spatial resolution is the issue, tractions decay with distance from the cells. In fig 5dii (left) (and 5eii left), tractions appear large with no noticeable decay at the center of the nonadhesive patch. However in the kymographs, tractions generally appear small in the center of the circle, suggesting that the image in 5dii (left) isn't representative of normal behavior. For how many data sets and how many time points is traction large inside the center of the adhesive patch? If only for a small number of images, this error, whatever its cause, may be negligible.
- The use of a line tension to summarize traction data and compare to the model could potentially improve the analysis, but there are many open questions:
1. It is unclear how the line tension is computed from the traction data. The only details I can find on this computation are line 273 ("radial traction force divided by radius of the extruding cell") and supplemental figure 11 ("radial traction force divided by the perimeter of the closing area"). Units of traction force are force / length^2, so units of traction/radius or traction/perimeter will be force / length^3. These units do not match units of line tension (force/length).
2. The equation on line 278 writes F_{radial} dr >= \gamma 2 \pi R dr, where F_{radial} is defined as the radial traction force and \gamma is previously defined as line tension. However, on line 273, the line tension is defined to be radial traction force divided by radius of the extruding cell, so it appears that there are two equations that look quite similar but have different meanings—on line 273 is a definition suggesting traction and purse string force area proportional and on line 278 is a condition used to interpret the results.
3. Text on line 277 gives interpretation of the equation on 278. The authors write "the radial traction force needs to be large enough to generate the work to overcome the resistivity of the bulk." However, isn't it possible that forces of the purse string could fully balance resistivity of the bulk with no traction? And if there is traction, doesn't the traction result from a portion of the forces produced by the purse string (since there are no lamellipodia next to the nonadhesive patches). Therefore, wouldn't a force balance imply that purse string tension = traction force + resistivity of the bulk? Instead of this, the text starting on line 277 suggests traction force = resistivity of the bulk and the equation on 278 suggests that traction force >= purse string tension.

4. supplementary fig 11 shows work, defined as F_{radial} dr. It is not stated how dr is determined; it appears that dr is computed from a time derivative of the graphs of radius of extrusion area, but in this case, the authors measure dr/dt, giving dW/dt, rather than W.
5. The equations are used to approximate the line tension of 6.5 nN/um on the largest adhesive patch. But as stated in comment 3, there seem to be 3 variables, line tension, traction force, contractility of the bulk. Can traction force and contractility of the bulk both be quantified so that line tension can be computed?

Reviewer #2 (Remarks to the Author):

The revised version of the manuscript "Adhesion-mediated heterogeneous actin organization governs apoptotic cell extrusion" by Phuong Le et al. is much improved and the authors have taken steps to significantly address the suggestions of the reviewers. To better clarify their message, important data from the supplementary figures has been moved to the main figures, and additional data has been added to the modified main figures to support their conclusions. The authors also now compare numerical simulations derived from their theoretical model with experiments using different α-catenin mutants seeded on different non-adhesive patch sizes to bring a more unified view of the factors contributing to completion of extrusion. In my opinion, the authors have adequately addressed the reviewers' critiques, and the article is now suitable for publication.

Reviewer #3 (Remarks to the Author):

In their revision, Phuong-Le &al have significantly improved their manuscript and answered to some of my comments. Some points are still not completely clear to me:
- In Fig. S11 and in lines 271-284 an evaluation of the tension in the purse-string is performed. I think there are some issues in Fig. S11 (dW is a differential and it is not clear how "dr" is calculated) but I am also wondering about the consistency between this description and the more general model described later? For instance, how would sigma_mu influence this analysis?
- Thanks to additional explanations I think I understand better the tension along the radial fiber cable; but it seems to me that to obtain Eq. 6 one needs to assume that the cable stays permanently basally anchored at its initial radius R0. I am wondering if this is not in contradiction with the picture of the tissue flowing as a fluid on the substrate?
- In general, I think that the model provides interesting results (I thought the existence of a threshold radius for successful extrusion was interesting) but is still not as strongly connected to experiments as, it seems to me, it could be. I thought it would have been interesting to compare model results with data e.g. in Fig. 1d (for the dynamics of contraction) or patterns of traction forces in Fig. 5e. However I agree with the authors that the model is bringing interesting insights in any case.

Reviewer #1 (Remarks to the Author):

After reading the revised manuscript and the rebuttal letter, the following questions remain:

Regarding the model:
We thank the referee for his interest in the modeling part.

- The main text describing the model has been revised, but there are still insufficient details in the main text. For example, the reader has to look to the supplemental information to see that the model assumes the cell layer is a viscous incompressible fluid. This assumption is important, because it is required to relate the stresses with the change in radius.

We thank the referee for raising this point. We now provide further information into the main text and added the following paragraph:

Our model is based on a description of the bulk tissue as a continuum, incompressible viscous material characterized by a homogeneous and isotropic viscous modulus (η). Such description is motivated by the large duration of the extrusion process compared to previous visco-elastic time estimates (1h time-scale in [Tili PRL 2020]).

- My questions about the importance of the model have been partly addressed in the rebuttal letter, including comments responding to a similar question by reviewer 3. In short, the model does two things. 1. It shows how purse string contraction and nonadhesive patch size jointly affect extrusion, with findings being as expected and therefore demonstrating that the model can capture some experimental observations. 2. It shows that fluctuations in contraction can increase the probability of extrusion, which is a new result that builds on experimental findings. In the current version of the manuscript, the results on stress fluctuations are located in supplemental information, and the text describes the fluctuations at the end of the section on the model rather than at the start. The importance of this result would be clearer if the manuscript were reorganized to draw more attention to the idea of fluctuations.

We agree that the idea of fluctuation induced forces is a rich subject, which we now briefly discuss in the main text – providing a further stronger bound between the experimental system and the theoretical framework.

Most mechanical studies interpret the final mechanical configuration of a system in terms of a static and deterministic approach. Yet, extrusion being an irreversible process, the extrusion probability (within a given observation time window) can sensitive to both the amplitude of stress fluctuations and their correlation time. Within a parameter range that is experimentally reasonable, our framework shows that both a larger amplitude (D_A) and a longer correlation-time (tau) help to reach the extrusion state ($R = 0$). This is consistent with expectations, e.g. a long-stochastic weakening of the strength of apico-basal fibers is expected to favour a successful extrusion. However, formally, dealing with finite correlation time leads can lead to complex analytical expressions for the extrusion rate probability. Here, we show that we can consider the effect of time-correlation through two limit cases that are analytical tractable:
(2a) the one where the force correlation time is given by the visco-elastic time of the tissue, hence modelled as delta-correlated (since the visco-elastic time is negligible at long time scales);
(2b) the one where the force correlation time is long compared to the visco-elastic time of the tissue, modelled as a exponentially-correlated active stress term.

Based on our previous theoretical work [Rupprecht et al. PRL 2018], we have shown that the dynamic equation differ in both case; long-time correlated stress fluctuations (even zero-averaged) generically lead to the emergence of an additional deterministic force (aka the Stratonovich spurious drift term) that can be shown to help the closure process; such force does not arise when a delta-correlated stress is considered. There, the interplay between the stress correlation time and the viscoelastic time could play a crucial role in the extrusion process. These considerations provide a further extension to previous treatments (Nier PNAS 2015 & Cochet-Escartin Biophys. J 2014).

Having reclarified this theoretical points in the new version of the manuscript and SI, we therefore reconsidered the experimental data to extract the amplitude and correlation of the radial force fluctuations, estimated through traction force measurement.

We now show that in α-catenin KO, the amplitude of fluctuations of radial stresses is higher than for wild-types (see SI Fig 13a-c). We therefore considered in our stochastic simulation framework a modulation of the amplitude of fluctuations; we did not observe a significant increase of the extrusion probability at low purse-string values (see SI Fig 13d-f) under a wide range of physically reasonable range of parameters. The additional observation that the correlation time of fluctuations is smaller in α-catenin KO than in the WT case further justifies that fluctuations, although crucial to understand the whole opening process, are not sufficiently large to completely dominate over deterministic forces.

We now write in the main text (page 15, line 386 – 409):

Being irreversible, stochastic fluctuations could drive the extrusion to completion even when average forces would prevent it. By considering a mechanical stress fluctuation term ξ in Eq.1, we show that fluctuations can crucially contribute to the statistics of the closure of the extrusion site (see also Supplementary Note - Eqs. 10 and 11). We used numerical simulation to solve the probability of extrusion as the function of initial size and probability according to this framework. We compared our simulations with experiments performed with cells expressing different αcat mutants (Fig.8e-f) on different non-adhesive patch sizes. The trend obtained from numerical solutions varying the strength of the purse-string contractility (see Fig.8e-f) closely follows the trend observed in experiments with different αcat mutant background for cells on non-adhesive patches. Our analytical model demonstrated that the presence of fluctuations may lead to the completion of the extrusion process even in a phase space of parameters where deterministic forces would prevent it from occurring.

We estimated the level of fluctuations from the autocorrelation of the temporal fluctuations in the averaged radial force. We found that stress fluctuations were higher for α-catenin KO than for wild-types (see SI Fig 13a-c); incorporating an increased level of fluctuations of αcat mutants in our stochastic simulation still lead similar extrusion probability as in Fig. 5 (see SI Fig 13d-f).

The assumption of a viscous bulk tissue undergoing fluid flows is not incompatible with the assumption of a constant pre-stress exerted by the apico-basal cables. In spite of an expected fast turnover time of focal adhesion (in the min time-scale), new focal adhesions are constantly formed within a specific region at the edges of the non-adhering substrate; similarly, in spite of an expected fast remodeling time of individual actin fibers (in the min time-scale), the apico-basal fibers can remain stable (e.g. with stable anchoring over the substrate) over the whole duration of the experiments.

Regarding the analysis of traction force:
- Nonzero traction forces are observed inside the nonadhesive patch. The rebuttal letter suggests that bead displacements inside the adhesive patch could cause nonzero tractions inside the patch. This is not necessarily true, as nonzero bead displacements in the center of the patch could exist even if no tractions

are located inside the patch. However, limited spatial resolution could potentially cause tractions to exist in a region slightly larger than the boundary of the cells. However, if spatial resolution is the issue, tractions decay with distance from the cells. In fig 5dii (left) (and 5eii left), tractions appear large with no noticeable decay at the center of the nonadhesive patch. However in the kymographs, tractions generally appear small in the center of the circle, suggesting that the image in 5dii (left) isn't representative of normal behavior. For how many data sets and how many time points is traction large inside the center of the adhesive patch? If only for a small number of images, this error, whatever its cause, may be negligible.

We thank the reviewer to point that out. In plotting the heat map for fig. 5dii and fig. 5eii, we took the average of all time points (before and after extrusion) for 3 sets of data. We realized that the time points before extrusion might contribute to no decay observed at the center of extrusion patch. Therefore, we re-plotted the heat map for fig. 5dii and fig. 5eii by considering only the time points after extrusion start time and as such, we observed the decay at center of non-adhesive patch, as shown in the fig.5 of revised version of the manuscript.

- The use of a line tension to summarize traction data and compare to the model could potentially improve the analysis, but there are many open questions:
1. It is unclear how the line tension is computed from the traction data. The only details I can find on this computation are line 273 ("radial traction force divided by radius of the extruding cell") and supplemental figure 11 ("radial traction force divided by the perimeter of the closing area"). Units of traction force are force / length^2, so units of traction/radius or traction/perimeter will be force / length^3. These units do not match units of line tension (force/length).

We agree with reviewer that this part was suffering of a lack of clarity. We have rewritten this paragraph to make it clear and consistent. We estimated the line tension from the measurements of the traction forces, T_r (in Pa), along the perimeter of the non-adhesive zone. To compute the radial force *Fr* (in N), we thus integrate the magnitude of the traction forces over a ring of radius R and 1 µm-width. As shown in the revised SI, the unit of the line tension γ in 2D is in N/m (at equilibrium, $\gamma = Fr/2\pi R$).

We made changes to the manuscript accordingly (P. 11-12, line 282-294):
"We estimated the tension contributed by purse-string using force measurement on non-adhesive patch of D = 30 µm. We represent the contractile force exerted by purse-string by the line tension γ (See Supplementary information, Theoretical appendix). On non-adhesive patch of D = 30 µm, we observed that total and radial forces diminished together with the re-opening of the closing area during extrusion (Fig. 5d-i & e-i, Supplementary Fig.11a). Focusing on the non-adhesive case of radius R without lamellipodia protrusion, we observed conditions under which the closing area could reopened determined by a zero velocity of the leading front (dr/dt =0, Supplementary Figure 11a). Under this condition, the mechanical equilibrium between the traction forces and the purse-string contraction allows estimating the line tension: $F_r = \gamma.2\pi R$ where the radial force Fr (in N) is computed from the magnitude of the traction forces (in Pa) over a ring of radius R and width of 1 µm (See Theoretical appendix). From these measurements, we thus estimated that the line tension was γ = 6.5 ± 1.4 nN/µm (a value consistent with the value reported in [41] (Supplementary Fig. 11a, middle panel)."

2. The equation on line 278 writes F_{radial} dr >= \gamma 2 \pi R dr, where F_{radial} is defined as the radial traction force and \gamma is previously defined as line tension. However, on line 273, the line tension is defined to be radial traction force divided by radius of the extruding cell, so it appears that there are two equations that look quite similar but have different meanings—on line 273 is a definition

suggesting traction and purse string force area proportional and on line 278 is a condition used to interpret the results.

Again, we thank the reviewer for his/her comments. We have modified the text in the revised version to provide a consistent definition of the line tension, γ (See point 1).

3. Text on line 277 gives interpretation of the equation on 278. The authors write "the radial traction force needs to be large enough to generate the work to overcome the resistivity of the bulk." However, isn't it possible that forces of the purse string could fully balance resistivity of the bulk with no traction? And if there is traction, doesn't the traction result from a portion of the forces produced by the purse string (since there are no lamellipodia next to the nonadhesive patches). Therefore, wouldn't a force balance imply that purse string tension = traction force + resistivity of the bulk? Instead of this, the text starting on line 277 suggests traction force = resistivity of the bulk and the equation on 278 suggests that traction force >= purse string tension.

We agree with the reviewer that the tension induced by the purse string should be equal to the traction forces and bulk resistivity. However, we used the same experimental system as Nier et al. PNAS 2015 (See SI page 2) where they did not observe any measurable tension in the monolayer after laser ablation. We therefore conclude that we could neglect at first order the bulk resistivity.

4. supplementary fig 11 shows work, defined as F_{radial} dr. It is not stated how dr is determined; it appears that dr is computed from a time derivative of the graphs of radius of extrusion area, but in this case, the authors measure dr/dt, giving dW/dt, rather than W.

We thank the reviewers to point that out. We indeed made a mistake in the units of the y axis and it should be *dW/dt*. We have modified the revised version of the manuscript, computed *dr/dt* and directly used a force balance equation. (Line 290 page 12).

5. The equations are used to approximate the line tension of 6.5 nN/um on the largest adhesive patch. But as stated in comment 3, there seem to be 3 variables, line tension, traction force, contractility of the bulk. Can traction force and contractility of the bulk both be quantified so that line tension can be computed?

We now think that both points 4 and 5 – which we agree were problematic issues -- are now addressed through a clarified framework consistent with the SI model. In the main text, Equation (XX), line – we now show how to related the traction force, apico-basal stress fibers contractility (mu) and line tension (gamma) through a deterministic force balance equation.

Here is the text we now add in the SI:

We now focus on the non-adherent case. As defined in the Method section, we estimate the overall radial force at the edge of the non-adherent patch as:

$$F_{\text{exp}} = \int_{R_0}^{R_0+\delta} r dr \int_0^{2\pi} d\theta \quad T_r(r,\theta), \tag{8}$$

where $T_r(r,\theta)$ is the radial projection of the measured traction field (expressed in Pa).

In our theoretical model, we consider a similar quantity defined in the limit a vanishing small patch $\delta \to 0$. In this limit, the only non-vanishing contribution to forces come from the apico-basal cables localized at the edge boundary,

which, based on the model defined Eq. (6), leads to the following expression for the total radial force:

$$F_{\text{theory}} = 2\pi R_0 \sigma_\mu^{(\text{non-adherent})} = 2\pi R_0 \times \mu \frac{R(t) - R_0}{\sqrt{h^2 + (R(t) - R_0)^2}}. \tag{9}$$

We point that $\sigma_\mu^{(\text{non-adherent})}$ is to be expressed in the units of a two-dimensional stress, i.e. Pa.m^1.

Experimentally, we are interested in the value of the traction force at a final steady-state configuration. Neglecting at first fluctuation terms, such steady state corresponds to the assumption of a local mechanical equilibrium between apico-basal radial forces and the orthoradial purse-string contraction. Whenever the purse-string value is large, we have $R(t) \propto R_0 \gg h = 3\mu\text{m}$, hence that:

$$F_{\text{theory}} = 2\pi R_0 \sigma_\mu^{(\text{non-adherent})} = 2\pi R_0 \mu = 2\pi\tau. \tag{10}$$

Assuming that $F_{\text{theory}} \approx F_{\text{exp}}$, we can estimate for the parameters μ and τ. The extracted value for τ and μ can be compared to the estimated line tension estimated in [4], where a similar quantity $F = 2\pi R_0 \gamma$ is defined, with $\gamma = 15\,\text{mJm}^{-2} = 15.10^{-3}\,\text{Nm}^{-1} = 15\,\text{nN}\mu\text{m}^{-1}$.

Reviewer #2 (Remarks to the Author):

The revised version of the manuscript "Adhesion-mediated heterogeneous actin organization governs apoptotic cell extrusion" by Phuong Le et al. is much improved and the authors have taken steps to significantly address the suggestions of the reviewers. To better clarify their message, important data from the supplementary figures has been moved to the main figures, and additional data has been added to the modified main figures to support their conclusions. The authors also now compare numerical simulations derived from their theoretical model with experiments using different α-catenin mutants seeded on different non-adhesive patch sizes to bring a more unified view of the factors contributing to completion of extrusion. In my opinion, the authors have adequately addressed the reviewers' critiques, and the article is now suitable for publication.

We thank the reviewer for his/her positive comments.

Reviewer #3 (Remarks to the Author):

In their revision, Phuong-Le &al have significantly improved their manuscript and answered to some of my comments. Some points are still not completely clear to me:
- In Fig. S11 and in lines 271-284 an evaluation of the tension in the purse-string is performed. I think there are some issues in Fig. S11 (dW is a differential and it is not clear how "dr" is calculated) but I am also wondering about the consistency between this description and the more general model described later? For instance, how would sigma_mu influence this analysis?

We agree with the reviewer that this part was unclear. We have significantly modified the text, which now reads (page 11, line 282):

"We estimated the tension contributed by purse-string using force measurement on non-adhesive patch of D = 30 µm. We represent the contractile force exerted by purse-string by the line tension γ (See Supplementary information, Theoretical appendix). On non-adhesive patch of D = 30 µm, we observed that total and radial forces diminished together with the re-opening of the closing area during extrusion (Fig. 5d-i & e-i, Supplementary Fig.11a). Focusing on the non-adhesive case of radius R without lamellipodia protrusion, we observed conditions under which the closing area could reopened determined by a zero velocity of the leading front (dr/dt =0, Supplementary Figure 11a). Under this condition, the mechanical equilibrium between the traction forces and the purse-string contraction allows estimating the line tension: F_r = γ.2πR where the radial force Fr (in N) is computed from the magnitude of the traction forces (in Pa) over a ring of radius R and width of 1 µm (See Theoretical appendix). From these measurements, we thus estimated that the line tension was γ = 6.5 ± 1.4 nN/µm (a value consistent with the value reported in [41] (Supplementary Fig. 11a, middle panel)."

We also clarified the relation how *sigma_mu* influences the measured line tension in the following new SI paragraph

We now focus on the non-adherent case. As defined in the Method section, we estimate the overall radial force at the edge of the non-adherent patch as:

$$F_{\text{exp}} = \int_{R_0}^{R_0+\delta} r dr \int_0^{2\pi} d\theta \quad T_r(r,\theta), \tag{8}$$

where $T_r(r,\theta)$ is the radial projection of the measured traction field (expressed in Pa).

In our theoretical model, we consider a similar quantity defined in the limit a vanishing small patch $\delta \to 0$. In this limit, the only non-vanishing contribution to forces come from the apico-basal cables localized at the edge boundary,

which, based on the model defined Eq. (6), leads to the following expression for the total radial force:

$$F_{\text{theory}} = 2\pi R_0 \sigma_\mu^{(\text{non-adherent})} = 2\pi R_0 \times \mu \frac{R(t) - R_0}{\sqrt{h^2 + (R(t) - R_0)^2}}. \tag{9}$$

We point that $\sigma_\mu^{(\text{non-adherent})}$ is to be expressed in the units of a two-dimensional stress, i.e. Pa.m^1.

Experimentally, we are interested in the value of the traction force at a final steady-state configuration. Neglecting at first fluctuation terms, such steady state corresponds to the assumption of a local mechanical equilibrium between apico-basal radial forces and the orthoradial purse-string contraction. Whenever the purse-string value is large, we have $R(t) \propto R_0 \gg h = 3\mu$m, hence that:

$$F_{\text{theory}} = 2\pi R_0 \sigma_\mu^{(\text{non-adherent})} = 2\pi R_0 \mu = 2\pi\tau. \tag{10}$$

Assuming that $F_{\text{theory}} \approx F_{\text{exp}}$, we can estimate for the apico-basal contractility μ and the purse-string line tension τ. The extracted value for τ and μ can be compared to the estimated line tension estimated in [4], where a similar quantity $F = 2\pi R_0 \gamma$ is defined, with $\gamma = 15\,\text{mJm}^{-2} = 15.10^{-3}\,\text{Nm}^{-1} = 15\,\text{nN}\mu\text{m}^{-1}$.

- Thanks to additional explanations I think I understand better the tension along the radial fiber cable; but it seems to me that to obtain Eq. 6 one needs to assume that the cable stays permanently basally anchored at its initial radius R0. I am wondering if this is not in contradiction with the picture of the tissue flowing as a fluid on the substrate?

We thank the referee for pinpointing this feature of our model. We now insist, within the main text, on the fact that there is indeed no contradiction between the fact that the bulk of the tissue flows as a fluid on the substrate and the existence of permanently anchored tensile cables. Rightly, the turnover time of focal adhesion and actin fibers (from seconds to tens of minutes) is significantly shorter than the time scale of the experiments (several hours) - which motivates our assumption of a bulk viscous cellular material. However, new focal adhesions are constantly formed at the edges of the non-adhering substrate - hence the memory of the location the non-adherent patch is never lost, and new apico-basal actin fibers are constantly formed at this specific location. The effect of permanently reforming contractile apico-basal cables results in force that resists the deformation induced by a purse string – resulting in an effective Kelvin-Voigt dynamics even though the bulk tissue properties are assumed to be viscous.

- In general, I think that the model provides interesting results (I thought the existence of a threshold radius for successful extrusion was interesting) but is still not as strongly connected to experiments as, it seems to me, it could be. I thought it would have been interesting to compare model results with data e.g. in Fig. 1d (for the dynamics of contraction) or patterns of traction forces in Fig. 5e. However I agree with the authors that the model is bringing interesting insights in any case.

We thank the reviewer for his/her positive comments on the modeling part. To further connect the experiments and the model, we have added a paragraph in the main text where we further justify how our model can provide a clear interpretation for the value of the traction force value in terms of purse string tension. Indeed, we find that the apico-basal stress fibers, connected to the substrate through focal adhesions, are supporting exerted by the contractile purse string tension.

REVIEWERS' COMMENTS

Reviewer #1 (Remarks to the Author):

The revised manuscript has addressed nearly all questions. In particular, the presentation of traction force data and the description of the calculation of line tension is much improved.

In my opinion, the section in the main text describing the theoretical model is still difficult to understand without reading the full section on the model in the supplemental information. The addition that the model treats the tissue as an incompressible fluid gives important details, but then the viscous modulus is defined with no equation in the main text that uses it. Similarly, variable xi is listed in the main text but not used in an equation in the main text. Some full paragraphs in this section are dedicated to supplementary figures and supplementary notes. This criticism is really more about style than substance, so it’s a relatively minor point.

One other point is related to my question 3, about the assumption that resistance of the bulk is negligible. This assumption is based on one manuscript (Nier et al 2015) that stated, without showing data, that no retraction was present upon laser ablation of cells spanning a nonadhesive patch. It would be preferable to base this assumption on published results, rather than an unpublished observation. However, this is a minor point that should not hold up final publication of this manuscript.

Reviewer #3 (Remarks to the Author):

In their revision the authors have answered to my comments and brought clarification.
I just have two suggestions (I think I do not need to see the manuscript again):
- it seems to me that the line tension is called gamma in the manuscript and tau in the supplementary
- in section 1.4 of the supplementary it seems to me that the authors assume P_c =0, I would suggest to clarify and justify this.

Reviewer #1 (Remarks to the Author):

The revised manuscript has addressed nearly all questions. In particular, the presentation of traction force data and the description of the calculation of line tension is much improved.

In my opinion, the section in the main text describing the theoretical model is still difficult to understand without reading the full section on the model in the supplemental information. The addition that the model treats the tissue as an incompressible fluid gives important details, but then the viscous modulus is defined with no equation in the main text that uses it.

We have added in the main text the dynamical equation for R(t) in terms of the local friction constant and tissue viscous shear modulus.

Similarly, variable xi is listed in the main text but not used in an equation in the main text. Some full paragraphs in this section are dedicated to supplementary figures and supplementary notes. This criticism is really more about style than substance, so it’s a relatively minor point.

We introduce the noise variable xi later within the text as an additional contribution to Equation 1. The role of the noise is theoretically rich and interesting, yet for clarity, we would rather prefer to leave it to the appendix.

One other point is related to my question 3, about the assumption that resistance of the bulk is negligible. This assumption is based on one manuscript (Nier et al 2015) that stated, without showing data, that no retraction was present upon laser ablation of cells spanning a nonadhesive patch. It would be preferable to base this assumption on published results, rather than an unpublished observation. However, this is a minor point that should not hold up final publication of this manuscript.

As the reviewer 1 rightly points out, we indeed followed the conclusions from Nier et al., whereby the bulk tissue pressure (associated to incompressibility) is found to provide a relatively minor contribution with respect to other forces.

We have introduced an additional discussion within the main text to strengthen our conclusion:

“Estimates for the absolute value of the pressure field are expected to be in the Pc = 10-100 Pa.micron = 0.01-0.1 nN.μm-1 range. The corresponding forces appear small (overall in the 1nN) compared to the forces associated to lamellipodia or purse string structures, whose strength can be estimated in the 10nN range based on the value of traction forces close to the extrusion site (see Fig. 5d).” (pg15, line 388-392).

Nevertheless, we insist that such an overall tissue pressure field (providing a constant radial force) cannot explain extrusion failures (which require an increasingly high levels of radial forces - as for the contractile apico-basal fibers).

Reviewer #3 (Remarks to the Author):

In their revision the authors have answered to my comments and brought clarification.
I just have two suggestions (I think I do not need to see the manuscript again):
- it seems to me that the line tension is called gamma in the manuscript and tau in the supplementary

We thank the reviewer for stopping these typos. We have corrected this typo in the manuscript and also in Figure 6. We used the notations as in our manuscript to avoid ambiguities; here sigma is a two-dimensional tissue stress; tau is the notation for line tension and does not have the units of a surface tension (typically denoted gamma).

- in section 1.4 of the supplementary it seems to me that the authors assume P_c =0, I would suggest to clarify and justify this.

As both reviewers rightly point out, we follow the conclusions from Nier et al., whereby the bulk tissue pressure (associated to incompressibility) is found to provide a relatively minor contribution with respect to other forces.

We have introduced an additional discussion within the main text to strengthen our conclusion:

"Estimates for the absolute value of the pressure field are expected to be in the Pc = 10-100 Pa.micron = 0.01-0.1 nN.μm-1 range. The corresponding forces appear small (overall in the 1nN) compared to the forces associated to lamellipodia or purse string structures, whose strength can be estimated in the 10nN range based on the value of traction forces close to the extrusion site (see Fig. 5d)." (pg15, line 388-392).

Nevertheless, we insist that such an overall tissue pressure field (providing a constant radial force) cannot explain extrusion failures (which require an increasingly high levels of radial forces - as for the contractile apico-basal fibers).
